# Investigating T Cell Immune Dynamics and IL-6’s Duality in a Microfluidic Lung Tumor Model

**DOI:** 10.1021/acsami.4c09065

**Published:** 2024-10-29

**Authors:** Parvaneh Sardarabadi, Kang-Yun Lee, Wei-Lun Sun, Amir Asri Kojabad, Cheng-Hsien Liu

**Affiliations:** † Institute of Nanoengineering and Microsystems, National Tsing Hua University, Hsinchu 30044, Taiwan, R.O.C; ‡ Division of Pulmonary Medicine, Department of Internal Medicine, Shuang Ho Hospital, Taipei Medical University, New Taipei City 235, Taiwan, R.O.C; § Division of Pulmonary Medicine, Department of Internal Medicine, School of Medicine, College of Medicine, Taipei Medical University, Taipei 110, Taiwan, R.O.C; ∥ TMU Research Center for Thoracic Medicine, Taipei Medical University, Taipei 110, Taiwan, R.O.C; ⊥ Pythia Biotech LTD., New Taipei City 23561, Taiwan, R.O.C; # Department of Hematology, School of Allied Medical Sciences, Iran University of Medical Sciences, Tehran 14535, Iran; ¶ Department of Power Mechanical Engineering, National Tsing Hua University, Hsinchu 30044, Taiwan, R.O.C; ∇ College of Semiconductor Research, National Tsing Hua University, Hsinchu 30044, Taiwan, R.O.C

**Keywords:** immune cells, tumor microenvironment, chemokine, interleukin 6, microfluidic biochip

## Abstract

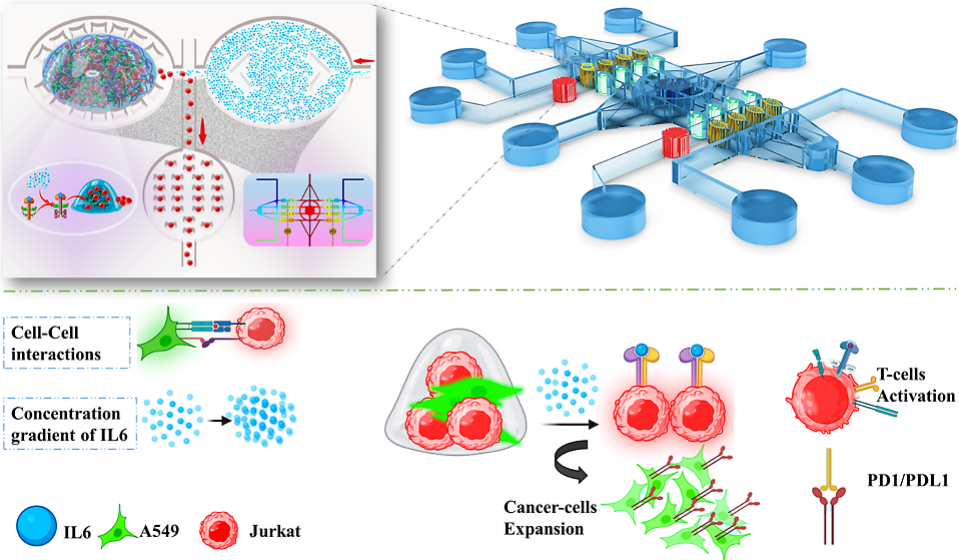

Interleukin 6 (IL-6), produced by immune cells, is crucial in promoting T cell trafficking to infection and inflammation sites, influencing various physiological and pathological processes. Concentrations of IL-6 and other cytokines and chemokines can influence T cell differentiation and activation. Understanding the dual faces of IL-6 within the tumor microenvironment is crucial to understanding its role. A flow-based microsystem was designed to investigate CD4^+^ T cell activation in response to different IL-6 gradients in an under-control 3D culture. The study found that cancer cells’ response to varying IL-6 concentrations was dynamic and dose-sensitive, with immune cell migration rates showing sensitivity to the IL-6 gradient. A549 cell expansion increases gradually and time-dependently with 50 ng of IL-6, while Jurkat cell migration follows a time-dependent pattern. However, when a total of 100 ng IL-6 concentration is applied, A549 cells expand rapidly, potentially influencing Jurkat cell migration. Jurkat cell mobility is lower, possibly due to increased A549 cell presence and heightened cell–cell interactions. Different IL-6 concentration gradients can modulate the expression of some CD markers like CD69 and programed cell death protein 1 in CD4^+^ T cells, suggesting that IL-6 concentration gradients affect immune cell phenotypes. This suggests that IL-6 plays a crucial role in activating T helper cells and may be involved in the later phases of inflammation. Also, the increased levels of IFN-γ and TNF-α highlight IL-6’s impact on T cell inflammatory response. This study emphasizes the intricate effects of IL-6 on T cell activation, phenotype, cytokine production, and phenotypic heterogeneity, providing valuable insights into immune response modulation in an experimental setting.

## Introduction

Lung cancer, with an 18% mortality rate in 2020, is one of the deadliest types of cancer.^1^ The complex relationship between the immune system and lung cancer cells in the tumor microenvironment (TME), historically viewed as a potential “magic bullet” for cancer treatment, has been found to involve inflammatory processes that can worsen outcomes.^2^ Chronic inflammation in malignant tumors stimulates cell infiltration and cytokine and mediator production, leading to an inflammation cycle. Inflammatory cells manage TME, contributing to tumor proliferation, tumor cell viability, and modulating metastasis.^3^ Key immune cells, including tumor-associated macrophages (TAMs), neutrophils, myeloid-derived suppressor cells (MDSC), and regulatory T CD4^+^ (Treg), drive neoplastic progression by secreting proinflammatory cytokines like tumor necrosis factor (TNF), transforming growth factor-β (TGF-β), interleukin-1 (IL-1), and interleukin-6 (IL-6).^4^ In the TME, IL-6, in conjunction with cytokines such as IL-1, IL-23, and TGF-β, intricately influences immune cell infiltration and function. IL-1 boosts IL-6 production and supports T helper (Th) cell differentiation, fostering inflammation and immune recruitment.^5^ IL-23 synergizes with IL-6 to maintain Th cells, enhancing the inflammatory response. TGF-β, while promoting Th differentiation with IL-6, also induces regulatory T cells (Tregs) and suppresses cytotoxic T cells and NK cells, balancing inflammation and immune suppression.^6^ IL-6 plays a critical role in maintaining the balance between proinflammatory Th cells and immunosuppressive regulatory T cells (Tregs), ultimately influencing immune cell infiltration and tumor progression.^7^ Understanding these dynamics is crucial for effective cancer therapy.^8^ Some cancers overexpress IL-6 or IL-11, activating pathways like PI3K-AKT-mTOR, NF-κB, RAS-RAF-MAPK, and STAT3, which promote epithelial-mesenchymal transition, cell proliferation, survival, migration, and cytokine production, fostering tumor growth.^9^ Proinflammatory cytokines can promote pro-tumorigenic inflammatory loops in cancers like lung cancer, enhancing cell proliferation, antiapoptosis signaling, angiogenesis, and metastasis, while Th1 cells play a crucial role in the adaptive immune system by coordinating phagocyte activation, antigen presentation, and the destruction of cancer cells.^10^ In several studies, IL-6 has been shown to modulate the CD4^+^T cell function. Through activation of Stat3, interleukin 6 (IL-6) promotes gene expression of a number of cytokines in CD4^+^T cells. Mitochondrial Stat3 enhances CD4^+^T cell motility and velocity through mitochondrial Ca2^+^-mediated signaling. IL-6 plays a pathogenic role in inflammatory diseases.^11^ The balance of immune cells and cytokines in the lung, disrupted by lung cancer, makes these elements key biomarkers for immunotherapy efficacy.^12^ In a clinical study examining various cytokines (IL-2, IL-4, IL-6, IL-10, IL-17A, TNF, and IFN-γ), only IL-6 showed a significant increase in the level of NSCLC patients. High levels of IL-6 are associated with increased lung cancer.^13^ Elevated IL-6 levels were associated with worse overall survival (OS) at 6, 12, and 24 months.^14^ Further research on IL-6 in independent cohorts could establish its role as a prognostic marker for NSCLC. Eitetsu Koh et al. found that IL-6 levels in tumor tissues and preoperative serum correlated with cancer staging and prognosis, normalizing postsurgery.^15^ Various studies indicate that IL-6 levels directly affect NSCLC prognosis, with higher levels associated with poorer performance status and progressive disease.^16,17^ Levels of IL-6 can promote tumor growth, survival, and metastasis as well as suppress antitumor immune responses. IL-6 also shows potential as a diagnostic marker in various cancers, including lung, oral, esophageal, and gallbladder carcinomas.^18,19^ The inflammatory process in TME, whether it is an early or chronic phase, alters the balance of chemokine concentration around the TME. Following, T cells differentiate into different T cell subsets with unique functions. CD69, a type II membrane protein and initial activation marker, facilitates effector T cell relocation and trafficking to the bone marrow (BM) upon TCR stimulation.^20^ The immune regulation role of programed cell death protein 1(PD-1) (CD279) plays a crucial role in controlling excessive T cell activation and limiting immune-induced tissue damage. Chronic TCR stimulation and PD-1 expression trigger T cell dysfunctionality, and cancer cells manipulate the PD-1 pathway to escape and resist immune responses.^10,21^ Here, we offer a TME modeling and cell–cell interaction to deepen our understanding of cell behavior in a TME via 3D culture in a lung TME chip. In this study, CD3, CD4, CD69, CD183 (CXCR3), CD196, and PD1/ programed death-ligand 1 (PDL1) markers have been targeted. Each marker indicates a specific state of T cells, revealing the nature and function of CD4^+^T cells. Additionally, we determined the levels of two pleiotropic inflammatory chemokines, IFN-γ and TNF-α as well. According to our findings, the chemokines IFN-γ and TNF-α were significantly overexpressed in the first 24 h; however, during the second 24 h, the levels of protein changed. Understanding the role of IL-6 at different concentrations and its interactions with cells in the TME can enhance the efficacy of cancer immunotherapy and improve patient outcomes.

## Methods and Materials

### Lung TME Chip Design

A polydimethylsiloxane (PDMS) chip containing symmetric microfluidic areas was designed for long-term cell culture. In this device, the depth of cell culture areas (CCCs) was 100 μm and the total width of each chamber was 800 μm. Several outlets and inlets were considered in this flow-based lab chip. IL-6 was constantly pumped to the chip by symmetric inlets at the same time to create a balance of the IL-6 distribution in the IL-6 pool. Two inlets designed for fresh medium and two symmetric inlets with four extended branches for a balanced cell distribution in cell culure areas (CCAs) were included. Two outlets were developed for collecting migratory cells during passing time, which were connected to cell collection chambers (CCCs). For prolonged cell culture and to avoid bubble trapping in CCAs, bubble trap chambers near the outlets were constructed. Additionally, in the IL-6 pool and empty chambers (ECs), different structures of pillars were examined to reduce the chance of bubble trapping in the central part (Movie S1). This chip can provide a chemical gradient and mimic the phenomenon of chemotaxis by permitting IL-6 dilution [IL-6 + 20% fetal bovine serum (FBS) (Gibco)] through parallel complex channels and chambers. CCCs to monitor migrated immune cells were located on either side of the chip. A simulation study using COMSOL Multiphysics for the microfluidic chip design was performed to verify the ability to create different IL-6 gradients in CCAs. In simulations, it was shown that the microfluidic chip could create distinct IL-6 gradients in individual chambers by diluting the IL-6.

### Lung TME Chip Fabrication

The flow-based chip was fabricated using an EVG620, soft lithography, and a SU-8 2035 as the photoresist. The pattern on the photomask would be printed on the photoresist by UV. Following the addition of PDMS monomer and hardener (Sylgard 184, Dow Corning Corporation, Midland, USA) in a ratio of 10:1 (w/w), the PDMS layer was baked at 65 °C for 60 min. The mold was pulled off after PDMS was cured. Then, O_2_ plasma was applied to bond the PDMS layer to the glass. The device was complete. Further studies used 75% EtOH and UV germicidal lamps to maintain sterile conditions within the chip for the cultured cells.

### Cell Preparation

A549 (human lung adenocarcinoma cell line) cells and Jurkat T (human leukemic T cell lymphoblast) cells were collected from the Shuang Ho Hospital (Taipei, Taiwan). Cells were cultured in RPMI 1640 medium (Roswell Park Memorial Institute, 22400105, Gibco, Waltham, MA, USA) supplemented with 10% FBS (A4766801, Gibco, Waltham, MA, USA) at 37 °C with 5% CO_2_. Frequency of passaging cells was usually performed every 2–3 days. To culture on a 96 well-plate, cells were seeded at a density of ∼5000 cells per well mixed with 30 μL of GelMA and exposed to 35s UV light. To ensure consistent initial conditions, cells were washed 2 times with PBS before incubation. We tested the viability of Jurkat cells within GelMA and found that Jurkat cells were more sensitive than A549 cells in this environment. The LIVE/DEAD assay showed that ≥84% of Jurkat and A549 cells remained viable over three days in 5% GelMA. This high viability indicates that the GelMA matrix effectively supports cell health in the standard medium (Figure S4A,B). Compared to 1:1 and 1:2 ratios, a 1:3 ratio of A549 to Jurkat cells was better suited for introducing the cell mixture into the chip using GelMA. In this setup, approximately 1 × 10^6^ cells/mL of A549 and 3 × 10^6^ cells/mL of Jurkat cells (1:3 ratio) were prepared for the experiment. This ratio was optimal due to the chamber design and the pumping mechanism, resulting in approximately 1500 to 2000 encapsulated cells per chamber. Jurkat cells encapsulated in 5% GelMA showed good viability on the biochip after 48 h, indicating that this setup supports immune cell survival and functionality, making it suitable for immune response studies. Similarly, A549 cells demonstrated a high viability (≥88%) on the biochip, suggesting that it was conducive to cancer cell survival and proliferation, with the setup also being useful for studying tumor progression and cell interactions (Figure S4C–E, Table S4).

### Active Recombinant Human IL-6 Protein

Active recombinant human IL-6 (catalog number RP00201) was diluted to concentrations of 50, 100, and 200 ng. IL-6 is a multifunctional cytokine that regulates cell proliferation and differentiation, crucial for immune responses and acute phase reactions. It is produced at inflammation sites and secreted into the blood, where it stimulates a transcriptional inflammatory response via IL-6 receptor alpha. IL-6 concentrations above 20 ng/mL were chosen for the study despite typical blood levels in healthy individuals ranging from 0 to 43.5 pg/mL, with a critical threshold of 35 pg/mL indicating increased mortality and ICU admission risk. Cancer patients often have much higher IL-6 levels in serum, with means of 993 pg/mL in oral, 813 pg/mL in lung, 960 pg/mL in gall bladder, and 381 pg/mL in esophageal cancer.^19^ The high IL-6 levels used in the study reflect the assumption that chemokine concentrations were much higher in tissues (the site of inflammation) due to factors such as biological barriers, diffusion, gradient origins, local production, and tissue retention. Chemokines released into the bloodstream were quickly diluted and distributed, resulting in lower concentrations compared to the localized tissue environments where they are produced.^22^ Cell viability of Jurkat and A549 cells in the presence of different concentrations of IL-6 was analyzed in plate assays. Cells in both standard and 20% FBS conditions were examined over 48 h in the presence of different IL-6 concentrations to highlight the effect of a higher FBS concentration (20%)^23^ on cell viability in conjunction with IL-6 exposure (Figure S5A–C, Table S5).

### Fluorescence Staining in Biochip

To visualize immune cell movement in the lung TME chip, Jurkat T and A549 were stained with red (CellTracker Red CMTPX Dye, C34552, Invitrogen, Massachusetts, USA) and green (CellTracker Green CMFDA Dye, C2925, Invitrogen, Massachusetts, USA), respectively. Two LIVE/DEAD cell imaging kits were utilized. A LIVE/DEAD cell imaging kit (LIVE/DEAD Viability/Cytotoxicity Kit, L3224, Life Technologies Corporation, Carlsbad, USA) was applied after 48 h of coculture, (green/red) emission wavelength range (517, 617 nm) and excitation wavelength range (494, 528 nm). The LIVE/DEAD Cell Viability Assay kit utilizes two fluorescent indicators: CellBrite Red (*E*_x_/*E*_m_ = 613/631 nm) for staining viable cells and the cell-impermeable DNA-binding dye Nuclear Blue DCS1 (*E*_x_/*E*_m_ = 360/450 nm) for staining dead cells with compromised membranes.

### Fluorescence Microscope

The Olympus BX 51 fluorescence microscope with a Nomarski filter allows differential interference contrast (DIC) microscopy with a 100 W quartz halogen light source applied. We recorded bitmap images with a digital camera (FPC-3L310OIII, Forever Plus, Taipei, Taiwan) equipped with Forever Plus software (Forever Plus Crop, Taipei, Taiwan). Different filters were applied for visualization (Table S1). Images were obtained from multiple fields of view to ensure comprehensive and representative data. In particular, five replicates were imaged for each chamber, encompassing three distinct areas per sample. The fluorescence images were input separately into the Fiji ImageJ program and quantified automatically (Table S3). Essential experimental details about image processing and analysis are outlined in (Table S6). The step-by-step ImageJ process for A549 cell counting, including image conversion, thresholding, watershed, and manual counting, is provided in (Figure S9).

#### Flow Cytometry

The monoclonal antibody (antihuman) list was utilized for flow cytometry immunophenotyping of cells as mentioned in Table S2. The flow cytometry was taken using “BD LSR Fortessa cell analyzer” at Division of Pulmonary Medicine, Shuang Ho Hospital.

## Enzyme-Linked Immunosorbent Assay

Human IFN-γ solid-phase sandwich ELISA kit (JHC4021, sensitivity 4 pg/mL) and human TNF-α solid-phase sandwich (KHC3012, sensitivity 1.7 pg/mL) ELISA kit were prepared. TNF-α and IFN-γ protein concentrations were measured in the medium collected over 24 and 48 h from CCC outlets. TNF-α and IFN-γ levels have been measured through the ELISA kit according to the manufacturer’s protocol by an ELISA plate reader.

### Experimental Setup

The experimental hardware setup consists of a syringe pump (KDS230, KD Scientific, Holliston, MA, USA) for constant flow perfusion and a fluorescence microscope (Olympus BX 51 fluorescence microscope) equipped with a digital camera (FPC-3L310OIII, Forever Plus, Taipei, Taiwan) incorporated with Forever Plus software (Forever Plus Crop, Taipei, Taiwan) to record a series of bitmap images. The analysis was done by using Fiji ImageJ, a public-domain image processing program. To cure GelMA prepolymer, an OmniCure S1500 UV lamp (Excelitas Technologies Corp., Massachusetts, USA) was utilized. The structure and pore size of GelMA were monitored by high-resolution thermal field emission scanning electron microscopy (National Tsing Hua University) using a Japan JEOL, JSM-7610.

### Biocompatible Gelatin Methacryloyl

At 40 °C,10% (wt/vol) gelatin (gelatin from porcine skin, Type A G2500, Sigma-Aldrich, Missouri, USA) was dissolved in 300 mL of Dulbecco’s phosphate-buffered saline (Gibco, Waltham, MA, USA) buffer. Afterward, 1 g of MA (methacrylic anhydride, Sigma-Aldrich, Steinheim, Germany) was added gradually to the solution and mixed for 3 h. For 7 days, the dialysis membrane containing the final solution was dispensed into 5 L of deionized water. The pH value of GelMA was rebalanced to 7.4. Finally, the GelMA solution was lyophilized at −80 °C^24^.

To create GelMA prepolymer, the freeze-dried 5% (wt/vol final) GelMA and 0.5% (wt/vol) photoinitiator (photoinitiator, I-2959, Sigma-Aldrich) were well-mixed in PBS at 37 °C and then exposed under UV light (320–500 nm) at a power of 8.6 mW/cm^2^ using an OmniCure S1500 UV lamp (Lumen Dynamics, San Francisco, CA, USA) for 35 s to achieve photo-cross-linking at RT.^25^ GelMA hydrogel has been visualized by using high-resolution thermal field emission scanning at ×200 magnification, 15 kV, and WD = 11.2 mm, sputtering times in the 90s, current of 20 Ma, and scale bars of 100 μm. Variations in the mixture concentration and time can alter the hydrogel pore size and mechanical properties. For the analysis of hydrogel porosity, 5% concentrations of GelMA were incubated for 0, 24, and 48 h in fresh RPMI medium and frozen at −80. The 3D structure was detected by a high-resolution thermal field emission scanning electron microscope (National Tsing Hua University) Japan JEOL, JSM-7610F. The mechanical properties of GelMA, including its biocompatibility, bioactivity, photo-cross-linking properties, and the swelling ratio, were thoroughly evaluated.^26^ This comprehensive assessment ensures confidence in the effectiveness of UV cross-linking to maintain cell viability and uphold the integrity of experimental results. 35s exposure to UV light was optimized for GelMA cross-linking within the biochip. This UV exposure duration was optimized based on the cell viability rate and GelMA stability within the chip. At lower UV power settings, the stability of GelMA was compromised, leading to its displacement under constant flow conditions. The swelling ratio (SR) indicated that the GelMA hydrogel had increased in weight during 48 h (Figure S3A–D).

### Lung TME Chip Operation

Following sterilization, the bottom side of the lung TME chip was covered with a black mask, with the exception of the CCA chambers. Jurkat and A549 cells were suspended in 5% GelMA. The syringe pump simultaneously injected 50 μL of liquid-form GelMA mixed with a 1:3 (A549: Jurkat T) cell ratio from each outlet. In the next step, the liquid-form GelMA and cells’ mixture was exposed to UV light for 35 s from the bottom side of the lung TME chip. The cells were washed with fresh medium once the power was turned off to remove excess cells and residual photoinitiator solvent. In the last step, the lung tumor chip was placed in an incubator, and IL-6 and fresh medium from different outlets were used for continuous perfusion of the lung tumor chip for 48 h.

In order to observe the interaction of A549 and Jurkat T cells and immune cell migration in the lung TME chip, the cells were cultured in the 3D environment with continuous perfusion of IL-6 and medium from separate and parallel inlets for 48 h. Meanwhile, to monitor Jurkat T cells’ mobility from the cell-embedded GelMA hydrogel in CCAs to CCCs in response to the different concentration gradients of IL-6, a fluorescence microscope was utilized (Movie S2).

### Statistical Analysis

Data were statistically analyzed by applying Graphpad Prism v10.0 software (Palo Alto, CA, USA). The one-way analysis of variance followed by paired *t*-tests was utilized to compare the groups. Experimental results were expressed as means ± standard deviations; *p* <0.05 was considered statistically significant. Analysis of flow cytometry data was performed by using FlowJo.

## Results and Discussion

### Construction of the Lung TME Chip

For the platform described below, we utilized GelMA as a 3D microenvironment system due to its microscale geometries and mechanical properties.^27^ The goal was to fabricate a lung TME chip with the feature of a concentration gradient generator chip lithographically through ideal chambers and channels that mimic immune cell interaction with cancer cells in the presence of different concentration gradients of IL-6 in TME. An appropriately constructed symmetric microfluidic chip-based PDMS includes several channels to create a dilution system to promote the formation of a chemokine gradient in a 3D structure. Also, the heart of the chip consists of CCAs and CCCs. The two inlets are symmetrical and considered for equal cell loading into the chip plus bubble trapping to reduce the chance of bubbles stuck in the culture area. Eight chambers in this lung tumor lab chip are separated by channels, and the CCAs contain the target cell population. GelMA, A549, and Jurkat cells were blended and introduced into CCAs in a ratio of (1:3). To keep the stable hydrogels inside the CCAs intact, we incorporated pillars into the CCAs.

**Figure 1:**
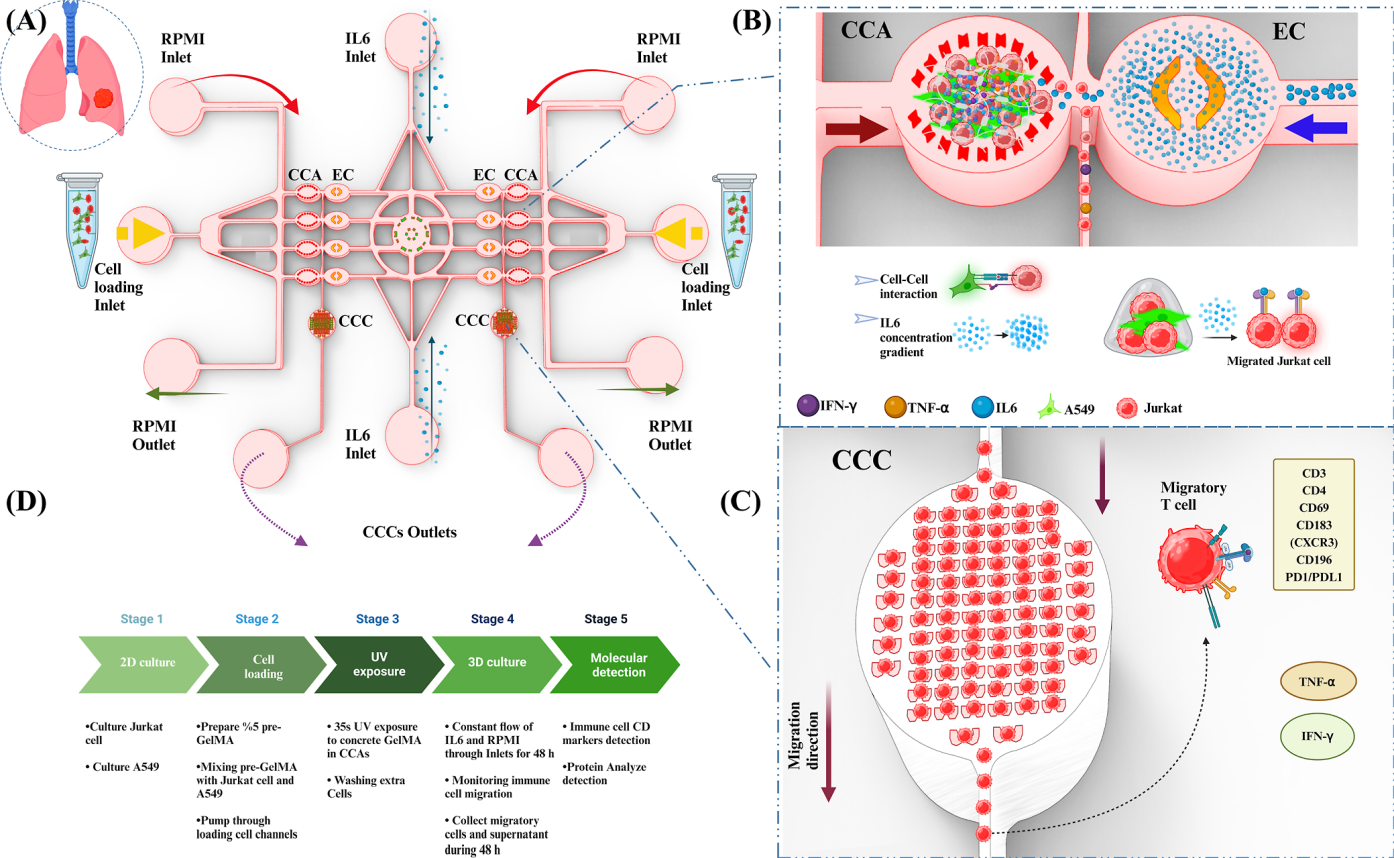
Overview of the lung TME chip operation principles. (A) A symmetric flow-based lung tumor lab chip includes several channels, an IL-6 pond in the center, and multiple areas for making different concentrations of IL-6 as well as the following: IL-6 inlets, cell collecting outlets, RPMI inlets, RPMI outlets, cell loading outlets, bubble trapping areas, CCAs for long-lasting cell culture, and CCCs with U-shaped pillars well designed. (B) A549 and Jurkat cells were mixed with pre-GelMA. Through cell-loading inlets, cells mixed with hydrogels were distributed onto the chip. Cells were loaded into chambers with a 1 × 10^6^ μm/min flow rate. The designed photomask was attached to the bottom side and UV light was irradiated for 35 s to synthesize GelMA’s polymer structure in CCAs. (C) After activating the IL-6 signaling pathway on T cells, activated T cells migrate from CCAs to ECs. However, due to the constant flow in biochips, migratory cells move through channels to CCCs. Supernatant and migrated cells were collected for 48 h from CCC outlets. The supernatant was analyzed for concentrations of IFNγ and TNF-α, as well as several CD markers CD4/CD8/CD69/CD183/CD196/PD1 targeted for future analysis. (D) In this diagram, all steps of biological experiments are summarized.

Furthermore, crescent pillars (U-shaped) were added to ECs to prevent bubble trapping. Two inlets for RPMI and two inlets for IL-6 for constant flow were designed. Depending on the desire to do experiments, four chambers are set aside for experiments, and another four are set aside as controls (Figures 1A) and S1A,B). The experiment’s cell movement mechanism is illustrated in (Figure 1B,C). By letting IL-6 dilution occur within chambers and central channels, a symmetric chip produces a chemical gradient. In order to activate the IL-6 signaling antitumor pathway, we will examine the pro-tumor effects of IL-6 in the coculture area. In order to collect migrated cells from the control and experimental chambers, two distinct CCCs are on either side of the chip. For this comprehensive assessment, flow cytometry was used to examine various cellular markers and characteristics. In addition, a systematically collected medium sample from the chip will be examined via ELISA to provide valuable information about the factors and cytokines secreted within the microenvironment (Figure 1D). Utilizing COMSOL Multiphysics, a physics interface study was undertaken to simulate diverse concentration gradients of IL-6, represented in mol/m^3^ (Figure S1C). The microsystem chip was fabricated through a soft lithography process with a 100 μm depth. Tubes with a 1.5 mm inner radius were placed on the silicon chip mold, and a 4 mm thick PDMS layer was fabricated. The PDMS layer was cured at 65 °C for 60 min and then O_2_ plasma was applied to bond it to the glass. The chip was primed with 75% ethanol in water and flushed with PBS (Figure S1D).

### Gelatin Methacrylate Hydrogel

GelMA is an organic hydrogel that is biocompatible and chemically adjustable. By adjusting the mixture concentration and passing time, the pore size and mechanical properties can be altered.^28^ The characteristics of a 3D culture can influence the rate of cell migration.^29^ To account for the potential impact of pore size changes over time, we conducted measurements to track the pore size evolution at various time points. To form the GelMA polymer, 5% of GelMA prepolymer was dropped on the glass and exposed to UV light for 35 s. First, sample 1 (0 h) was frozen and lyophilized at −80 °C for 24 h, while samples 2 (24 h) and 3 (48 h) were incubated in fresh medium for 24 and 48 h, respectively. Lyophilization was performed as with sample 1 (0 h). The pore size of GelMA hydrogel was determined with a scanning electron microscope (Figure S2A). The results showed that the size of the pores would change during the passing time in comparison with the control (sample 1). Since pores change in size over time, the suggested concentration of 5% of GelMA might affect the rate of cell migration^28,30^ (Figure S2B,C).

### Dilution of 50 ng IL-6 in the Gradient Generator Chip

**Figure 2:**
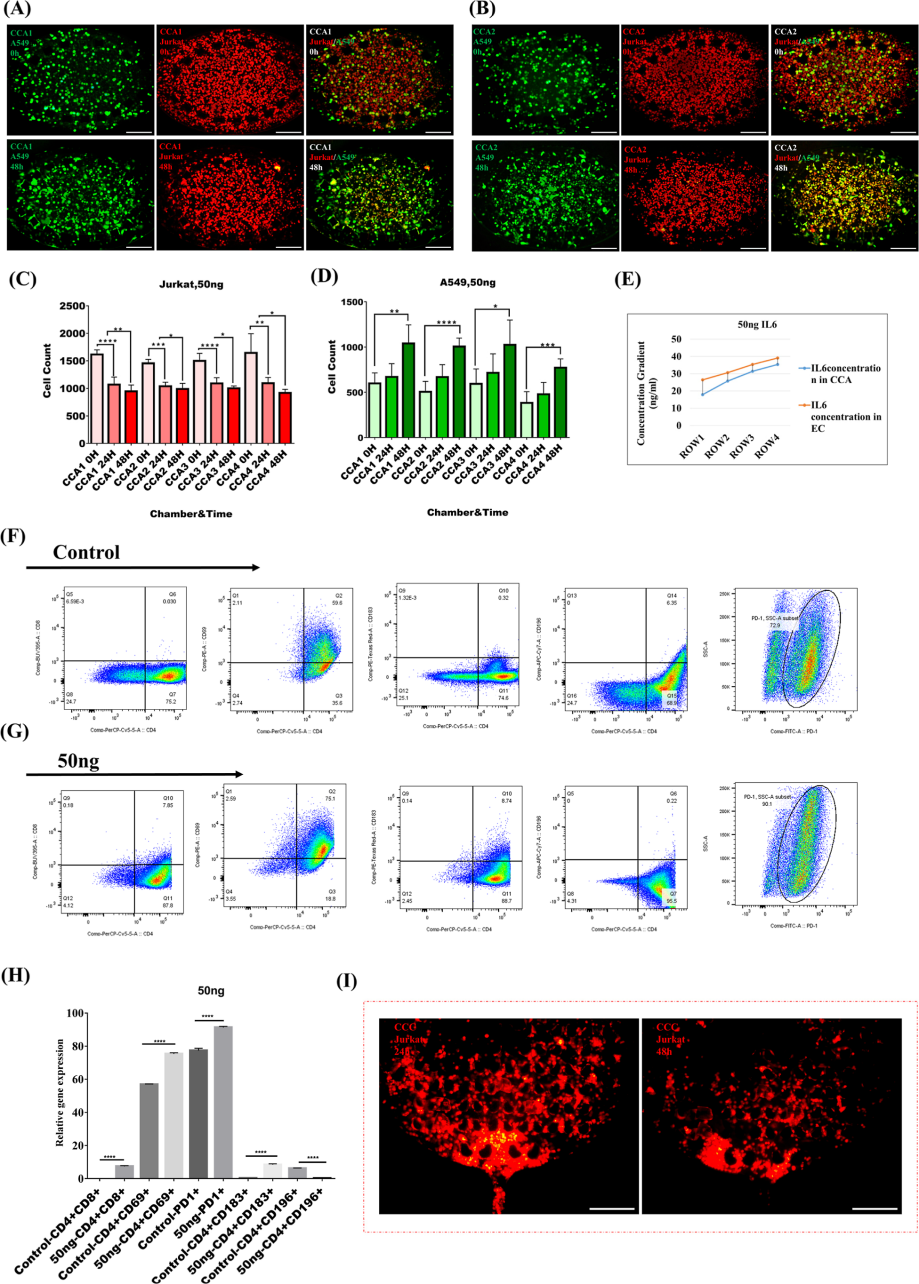
Effects of diluted 50 ng pumped IL-6 into the lung TME chip on cancer cells and immune cells cocultured. Jurkat cells are stained in red (CellTracker Red CMTPX dye) and A549 in green [CellTracker Green CMFDA dye (Invitrogen)]. (A) CCA1, coculture of A549 (green)/Jurkat (red), observation in 0–48 h. (B) CCA2, coculture of A549 (green)/Jurkat (red), observation in 0–48 h. The scale bar is 200 μm. Statistical analysis of Jurkat cell migration and A549 cell proliferation in CCAs. (C) The number of Jurkat cells and (D) A549 encapsulated in hydrogel during 0, 24, and 48 h in all chambers was counted by ImageJ and analyzed by GraphPad Prism 10. ****p <0.0001, **, *p* <0.001, *p <0. 05, ANOVA was used to compare 3 groups and the *t*-test was used to compare Jurkat cell groups. (*n* = 5). (E) IL-6 ratio concentration gradients are simulated by COMSOL in the lung TME chip chambers, while 50 ng IL-6 is pumped through two inlets at the same time. (F) Flow cytometry data. A number of monoclonal antibodies (antihuman) were used to assess the immune phenotypic characteristics of Jurkat cells. For further characterization, Jurkat cells were cocultured with A549 in the lung TME chip in the presence of different concentration gradients of a total of 50 ng IL-6 for 48 h. Then, Jurkat cells were obtained from CCC outlets in the experimental group. The control group was stained for multiple surface markers to characterize CD4/CD8/CD69/CD183/CD196/PD1 markers on Jurkat cells without the presence of IL-6. For gate, cell debris, doublets and dead cells have been omitted from the analysis. (G) Illustration of expression percentage of CD4/CD8/CD69/CD183/CD196/PD1 in the experiment group (50ng IL-6). (H) The percentages of cells expressing CD4/CD8/CD69/CD183/CD196/PD1 (*n* = 5) are shown for each cell subset: mean ± SEM *t*-tests were used to compare Jurkat cell CD markers between the control and the experiment groups. ****p <0.0001, **, *p* <0.001, *p <0.05. (I) In the CCC, migrated Jurkat cells (CellTracker Red CMTPX dye) have been detected for 24–48 h. The scale bar is 200 μm.

IL-6 has been confirmed in recent decades as a pleiotropic cytokine that regulates immune response, inflammation, hematopoiesis, and the nervous and endocrine systems.^31^ The responses of immune cells (Jurkat T) and cancer cells (A549) to different concentration gradients of IL-6 were explored in our tumor lab chip that constructed the in vitro cancer-immune microenvironment. 50 ng IL-6 + 20% FBS (Gibco) was pumped to the tumor lab chip for 48 h. We monitored the effect of IL-6 as a polytropic chemokine on T cell mobility and cancer cell proliferation. Jurkat cells are stained in red (CellTracker Red CMTPX dye) and A549 in green [CellTracker Green CMFDA dye (Invitrogen)], respectively. GelMA was mixed with A549 cells (green) and Jurkat cells and then injected into the lung tumor lab chip through two cell loading outlets in the chip with a 1:3 (A549: Jurkat T) cell ratio. Jurkat T cells (red) and A549 (green) embedded in GelMA hydrogel at 0 h are shown in the first row of the imaging results. Jurkat T cells encapsulated in GelMA hydrogel decreased in number over 24 h and in a dose-dependent manner for 48 h. The rate of cell migration in the first 24 h (*P* <0.0001) was higher than that in the second 24 h (*P* <0.002). The merged images demonstrate that Jurkat T cells have a significant reduction in GelMA hydrogel in CCA1. The first row shows the number of A549 (green), while the second row shows an increase of A549 proliferation during 48 h (*P* <0.002), significant (*P* <0.05). As shown in the results, both A549 and Jurkat cells showed a change in population size in other chambers. This also suggests that the modified conditions in the cell environment affected cell growth in A549 as well as Jurkat cell migration toward a high concentration of IL-6 in a gradient-dependent manner. The cells responded differently to the modified conditions, with A549 cells showing an increase in population size and Jurkat cells showing a decrease in population size. This indicates that cells can migrate and proliferate in response to the chemical gradients in the chamber (Figure 2A). In CCA2, there was significant cell number mobility during 24 h (*P* <0.001) and the rate of cell migration slowed in the second 24 h (*P* <0.028). The rate of A549 proliferation was significantly high (*P* <0.0001) (Figure 2B). Accordingly, in chamber CCA3, cells responded clearly to the IL-6 gradient in a cocultured A549 (green)/Jurkat (red) 3D culture. Data analysis shows Jurkat cell migration in 0 to 24 and 24 to 48 h to be (*P* <0.0001) and (*P* <0.038), respectively. And, A549 proliferation was (*P* <0.027) (Figure S6A). In CCA4, location chamber at the bottom, coculture of A549 (green) and Jurkat (red), Jurkat cells reduction in 0 to 24 h (*P* <0.008) was faster than 24 to 48 h (*P* <0.01). There was a significant increase in A549 proliferation in this chamber by (*P* <0.001) (Figure S6B). The charts show the quantification of Jurkat cell migration and A549 cell proliferation in CCAs. Jurkat and A549 cells were encapsulated in GelMA in all chambers. Over time and with varying doses, cells exhibited distinct and specific responses in each chamber that were easily distinguishable (Figure 2C,D). Jurkat cells exhibited enhanced migration toward IL-6 gradient concentrations, especially within the first 24 h, suggesting that moderate IL-6 levels stimulate immune cell migration and may reflect an active immune response within the microenvironment. Meanwhile, A549 cells showed increased proliferation over 48 h, with a marked acceleration during the second 24 h, indicating that the dilution gradient generated from 50 ng/mL of IL-6 in the biochip promotes cancer cell growth. This underscores the dual role of IL-6 in both driving immune cell migration and facilitating tumor progression. In order to determine the expression of CD4/CD8/CD69/CD183/CD196/PD1 markers on (Jurkat) T cells, two control groups were considered. The monoculture of Jurkat cells in the presence of multiple concentrations of IL-6 in a 2D culture (Figure S7A–E) was analyzed. Also, PD1/PDL1 in the A549 population as the control group was monitored (Figure S8B). To mimic chemotaxis phenomena in the biochip, COMSOL Multiphysics was utilized to simulate biochip functionality. A physics interface study was performed to simulate the IL-6 concentration gradients in mol/m^3^. Based on the design of the study, it is expected that ECs will possess a higher concentration of IL-6 than CCAs. IL-6 concentration gradient ratios in ROW1 (CCA1: 17.9 ng/mL, EC1: 26.4 ng/mL), ROW2 (CCA2: 25.8 ng/mL, EC2: 30.7 ng/mL), ROW3 (CCA3: 31.4 ng/mL, EC3: 35.4 ng/mL), and ROW4 (CCA4: 35.4 ng/mL, EC4: 39 ng/mL), respectively, were measured (Figure 2E). The following is the level of CD marker expression in the control group. 0.03% of the population CD4^+^CD8^+^, ∼57% CD4^+^CD69^+^, ∼0.32% CD4^+^CD183^+^, ∼6.3% CD4^+^CD196^+^, and a heterogeneous population were detected and ∼77.2% of them expressed PD1 (Figure 2F). As a main part of the study, we analyzed migratory cells isolated from CCC outlets to determine whether the mentioned markers are present on the surface of 3D-cultured (Jurkat) T cell subpopulations. In the experiment group, CD markers were expressed at the following levels: ∼7.8% of the population CD4^+^CD8^+^, ∼75% CD4^+^CD69^+^, ∼8.7% CD4^+^CD183^+^, ∼0.22% CD4^+^CD196^+^, and a large population ∼91% expressed PD1. All Jurkat cells from the control and experimental groups were collected and analyzed by flow cytometry for CD marker identification (Figure 2G). Data analysis of the CD marker’s expression between control and experiment groups revealed significant increases in CD marker expression in the experiment compared with the control (Figure 2H). Also, as a part of the study, the expression of PDL1 on Jurkat cells was analyzed (Figure S8A). There will be a gradual decrease in the number of migratory T cells that migrate in response to IL-6 over time. These cells migrated from CCAs in response to a gradient of IL-6. However, due to the medium flow inside the lung tumor lab chip, they were guided toward CCCs. Migratory cells are temporarily captured in CCCs by designed pillars (Figure 2I).

### Dilution of 100 ng IL-6 in the Gradient Generator Chip

**Figure 3:**
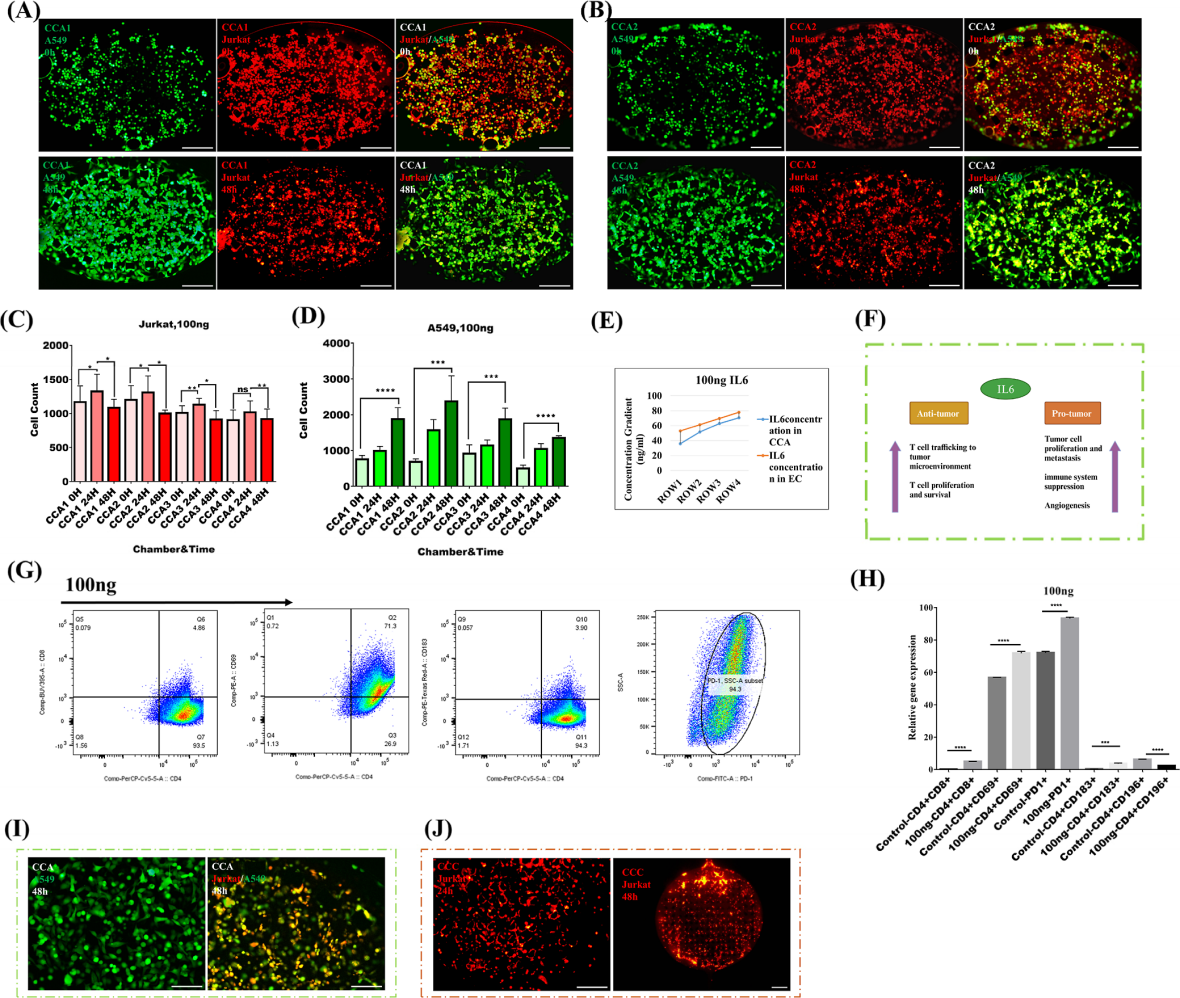
Analysis of A549/Jurkat cells cocultured in the lung TME chip for 48 h with IL-6 (100 ng) dilution. Jurkat cells are labeled in red (CellTracker Red CMTPX dye) and A549 in green (CellTracker Green CMFDA dye (Invitrogen)]. (A) CCA1, coculture of A549 (green)/Jurkat (red), incubation for 0–48 h. (B) CCA2, coculture of A549 (green)/Jurkat (red), observation for 0–48 h. The scale bar is 200 μm. The statistical analysis of Jurkat cell migration as well as Jurkat cell numbers embedded in hydrogel and A549 cell proliferation in CCAs. (C) The number of Jurkat and (D) A549 cells encapsulated in hydrogel during 0, 24, and 48 h in all chambers was counted by ImageJ and analyzed by GraphPad Prism10. ****p <0.0001, **, *p* <0.001, *p <0. 05. ANOVA was used to compare A459 3 groups and the paired *t*-test was used to compare Jurkat cell groups. (*n* = 5). (E) A COMSOL simulation was used to simulate IL-6 ratio concentration gradients in lung tumor chip chambers while 100 ng IL-6 was pumped through two inlets simultaneously. (F) Pro-tumor and antitumor illustration of IL-6. (G) Flow cytometry data. For further characterization, Jurkat cells were cultured in the lung TME chip in a total of 100 ng IL-6 and cocultured with A549 for 48 h after collecting from CCC outlets. Several monoclonal antibodies (antihuman) were used to identify the immune phenotypic characteristics of migratory Jurkat cells. Graphs illustrate the percentage of CD4/CD8/CD69/CD183/CD196/PD1 expression markers on Jurkat in the experiment group (100 ng IL-6). (H) The analysis of gate has excluded cell debris, doublets, and dead cells. Percentages of cells expressing CD4/CD8/CD69/CD183/PD1 (*n* = 5) are shown: mean ± SEM *t*-tests were used to compare Jurkat cell CD markers between the control and the experimental groups. ****p <0.0001, **, *p* <0.001, *p <0. 05. (I) Illustration of the coculture A549 (green) and Jurkat (red) after 48 h in the CCA. The scale bar is 100 μm. (J) It has been detected that Jurkat cells (CellTrackerTM Red CMTPX dye) have migrated directionally toward the CCC during 24 and 48 h in response to IL-6. The scale bar is 200 μm in 24 and 48 h.

Previous investigations have revealed that the levels of IL-6 and TNF-α in the serum of NSCLC patients diagnosed with lymph nodes or distant metastases were considerably higher than in cases without metastasis. IL-6 and TNF-α boost the proliferation and differentiation of NSCLC as well as stimulate tumorigenesis by modulating signaling pathways along with metastasis by facilitating epithelial-mesenchymal transitions of lung cancer cells, which contributes to the change in metabolism and resistance to chemotherapy and recurrence of tumors.^32^ In this step, we examined a total of 100 ng of IL-6 + 20% FBS (Gibco) to analyze multiple gradients with higher concentrations of IL-6. Jurkat cells (CellTracker Red CMTPX Dye) and A549 [CellTracker Green CMFDA dye (Invitrogen)] plus GelMA were inserted into the lung TME chip with the same ratio of cells of 1:3 (A549: Jurkat). The first row of figures shows the Jurkat T cells (red) and A549 (green) embedded in GelMA hydrogel at 0 h. The second row indicates that the number of Jurkat T cells encapsulated in GelMA hydrogel slightly increased over 24 h (*P* <0.02) and then gradually decreased over the next 24 h (*P* <0.027). Meanwhile, A549 showed different manners. The number of A549 sharply increased during the second 24 h (*P* <0.0001) (Figure 3A). CCA2-encapsulated cells also showed a significant increase in A549 counts. A549 proliferation and expansion were monitored for 48 h (*P* <0.0003). In addition, there was a slight increase in the frequency of Jurkat cells during 24 h (*P* <0.015). However, the number of Jurkat cells dropped in the second 24 h (*P* <0.04) (Figure 3B). In CCA3-encapsulated cells, a notable expansion in the A549 cell was monitored (*P* <0.0001). Jurkat cells were counted during 0–48 h and data analysis demonstrated (*P* <0.008) and (*P* <0.04), respectively (Figure S6C). In CCA4-encapsulated cells, an impressive growth in the A549 cell can be observed (*P* <0.0001) within 48 h. Jurkat cells were counted during 0–48 h, and data analysis demonstrated no significant variation in cell numbers within 24 h. However, Jurkat cells showed decreased numbers the following day (*P* <0.009) (Figure S6D). Statistical analysis of A549 proliferation and the impact of IL-6 on Jurkat cells is presented in Figure 3C,D. Jurkat T cells initially showed a slight increase in numbers within the first 24 h inside the chambers, but this was followed by a marked decrease in the subsequent 24 h, indicating that after 24 h, T cell migration occurred, leading to an increase in the number of migratory T cells. In contrast, A549 cancer cells exhibited a sharp increase in proliferation, particularly noticeable in the second 24 h, with significant growth observed throughout the experiment. This suggests that varying IL-6 levels promote cancer cell expansion, highlighting the complex role of IL-6 in modulating tumor-immune interactions within the microenvironment. 100ng/mL can be diluted in the lung tumor chip through the unique design and constant flow. COMSOL results show the concentration of IL-6 in each chamber as follows: IL-6 concentration gradient ratios in ROW1 (CCA: 35.7 ng/mL, EC1: 52.8 ng/mL), ROW2 (CCA2: 51.5 ng/mL, EC2: 61.3 ng/mL), ROW3 (CCA3: 62.9 ng/mL, EC3: 69.8 ng/mL), and ROW4 (CCA4: 70.7 ng/mL, EC4: 78 ng/mL) (Figure 3E). This diagram demonstrates and emphasizes the role of IL-6 as pro-tumor and antitumor faces of IL-6 in the TME (Figure 3F). The following results show the percentage of cells expressing different CD markers: 4.9% of the population are CD4^+^CD8^+^ cells, approximately 71.3% are CD4^+^CD69^+^, 3.9% are CD4^+^CD183^+^, 2.4% are CD4^+^CD196^+^as mentioned in Figure S8C, and roughly 94.2% of the population expresses PD1(Figure 3G). CD markers’ expressions were compared using an unpaired *t*-test (Figure 3H). The concentration of roughly 35.8–70.7 ng/mL IL-6 is associated with a significant expansion of A549 cells. In addition, it is impacted by the constant gradient in the chip between ECs and CCAs (Figure 3I). During the transition from CCAs in response to the gradient concentration, migratory T cells reside in CCCs temporarily (Figure 3J).

### Dilution of 200 ng IL-6 in the Gradient Generator Chip

**Figure 4:**
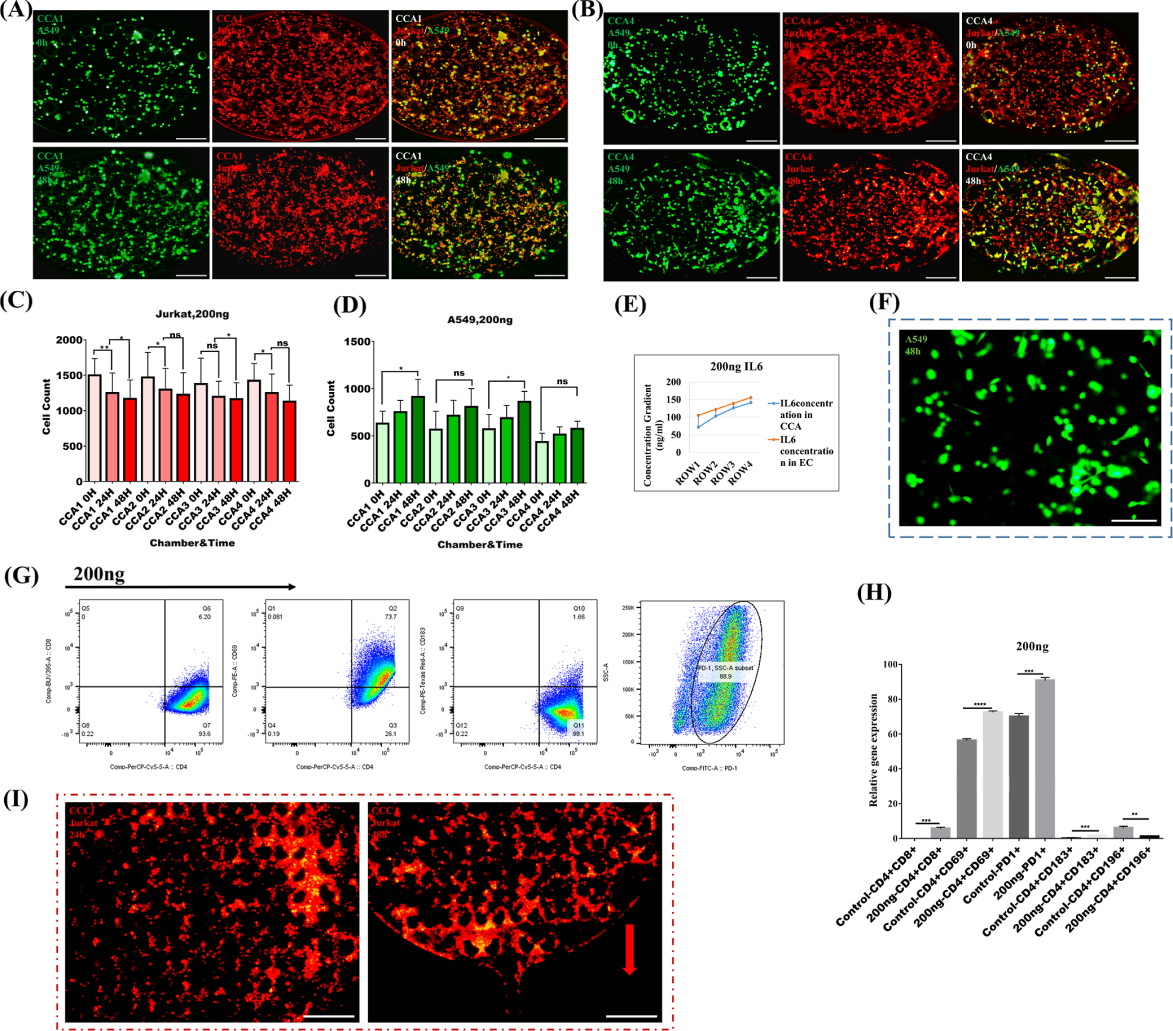
This data examine the results of coculturing A549/Jurkat cells with IL-6 (200 ng) dilution for 48 h on the lung TME chip. Jurkat cells are fluorescently dyed in red (CellTracker Red CMTPX dye) and A549 in green [CellTracker Green CMFDA dye (Invitrogen)]. (A) CCA1, coculture of A549 (green)/Jurkat (red), the first row of figures corresponds to 0 h and the second row corresponds to 48 h. (B) CCA4, coculture of A549 (green)/Jurkat (red), the first row of figures corresponds to 0 h and the second row corresponds to 48 h. The scale bar is 200 μm. Charts illustrate the migration and quantity of Jurkat cells embedded in hydrogel along with the proliferation of A549 cells in CCAs. (C) The quantity of Jurkat cells and (D) A549 encapsulated in hydrogel during 0, 24, and 48 h in all chambers was calculated with ImageJ and analyzed using GraphPad Prism 10. ****p <0.0001, **, *p* <0.001, *p <0. 05. ANOVA was used to compare A459 3 groups and the paired *t*-test was used to compare Jurkat cell groups. (*n* = 5). (E) A COMSOL simulation was utilized to model IL-6 ratio concentration gradients in lung tumor chip chambers under simultaneous injection of 200 ng of IL-6 through two inlets. (F) The figure shows the effect of IL-6 after 48 h on A549 (green) growth and expansion; meanwhile, A549 were cultivated with Jurkat cells in CCA. The scale bar is 100 μm. (G) As shown in the following charts, the percentage of CD4/CD8/CD69/CD183/PD1 marker expression on Jurkat cells that surface in the experiment group (total 200 ng IL-6) was analyzed by FlowJo. (H) The graph shows the comparison of expression levels of CD4/CD8/CD69/CD183/CD196/PD1 between the control group and experiment group (after harvesting from CCC outlets) (*n* = 5): mean ± SEM *t*-test. ****p <0.0001, **, *p* <0.001, *p <0.05. (I) Jurkat cells (CellTrackerTM Red CMTPX dye) have migrated toward CCC in response to IL-6 directionally within 24 −48 h. The scale bar is 200 μm.

In the final analysis, we examined the highest concentrations of IL-6 in TME in terms of its pro-tumor and antitumor properties. Jurkat cells (CellTracker Red CMTPX dye) and A549 [CellTracker Green CMFDA dye (Invitrogen)] along with GelMA were injected into the lung tumor chip in a 1:3 ratio (A549: Jurkat T). The first row displays Jurkat T cells (red) and A549 (green) embedded in GelMA hydrogel at 0 h. The second row demonstrated that the number of Jurkat T cells encapsulated in GelMA hydrogel gradually dropped over 24 h (*P* <0.002) and then minimally decreased over the following 24 h (*P* <0.017). Additionally, there was a slight increase in the A549 population during the second 24 h (*P* <0.037) as shown inFigure 4A. Interestingly, CCA2-encapsulated A549 cells did not show significant expansion within 48 h. However, the number of Jurkat cells in this chamber slightly decreased (*P* <0.012) in the first 24 h and no significant changes were observed during the second 24 h (Figure S6E). In CCA3-encapsulated cells, A549 cells gradually elevated (*P* <0.017). Accordingly, Jurkat cells did not show significant changes during the first 24 h, but in the second 24 h, the number of cells decreased slightly (*P* <0.047) (Figure S6F). Lastly, A549 cells did not display any significant changes. However, Jurkat cells showed a decrease in number over the first 24 h (*P* <0.022) (Figure 4B). Proliferation of A549 cells and IL-6 impact on Jurkat cells were statistically analyzed (Figure 4C,D). At the highest concentration, the dilution gradient generated from 200 ng/mL of IL-6, Jurkat T cells exhibited a substantial decline in migration over 48 h, with a more pronounced decrease in the initial 24 h (*P* <0.002) and a continued reduction in the following 24 h (*P* <0.017). This suggests that very high IL-6 concentrations may hinder immune cell migration, potentially impairing immune surveillance and response. In contrast, A549 cancer cells showed relatively limited proliferation, with only a slight increase in population during the second 24 h (*P* <0.037). This indicates that extremely high IL-6 levels might saturate the system, limiting further tumor cell growth. The highest concentration (200 ng/mL) was diluted in the lung TME chip. Due to the lung tumor lab chip design and constant flow, each chamber senses different concentrations. The COMSOL results show IL-6 concentration gradient ratios in ROW1 (CCA: 71.5 ng/mL, EC1: 105.7 ng/mL), ROW2 (CCA2: 102.9 ng/mL, EC2: 122.6 ng/mL), ROW3 (CCA3 125. Eight ng/ml, EC3: 139.5 ng/mL), and ROW4 (CCA4:141.4 ng/mL, EC4: 156.1 ng/mL) (Figure 4E). There was a low expansion rate of A549 cells in coculture with Jurkat cells in the presence of a high concentration gradient of IL-6 (Figure 4F). Here are the percentages of cells expressing distinct CD markers: 6.20% of the population are CD4^bright+^CD8^dim+^ cells, roughly 73.7% are CD4^+^CD69^+^, 1.66% are CD4^+^CD183^+^, 1.03% are CD4^+^CD196^+^ (Figure S8D), and 88.9% of the population are PD1^+^ (Figure 4G). The unpaired *t*-test is shown in Figure 4H. Migratory Jurkat cells (red) were short-term captured in CCCs over 0–48 h (Figure 4I).

### CD4^+^CD8^+^ DP T Cells (Double Positive)

Human CD4^+^CD8^+^ DP T cells (double positive) that express the tissue-homing marker CXCR3 were isolated from the blood. Our study detected a small heterogeneous population of CD4^bright+^CD8^dim+^ T cells among migratory cells. Understanding DP T cells’ migratory abilities is crucial for understanding their role in immune responses. DP CD4^bright+^CD8^dim+^ cells are activated and exhausted, releasing IFNγ and TNF, and have immune-regulatory properties that induce Th2 polarization and suppress Th1 in urological tumors.^33,34^ DP T cells (CD4^+^CD8^+^) with overexpression of PD1 and PDL1 impair the immune response against tumors by contributing to immune evasion and T cell exhaustion. They are critical targets for immune checkpoint inhibitors such as pembrolizumab and nivolumab, which restore T cell function by blocking the PD1-PDL1 pathway and can serve as predictive biomarkers for ICI response. Elevated circulating DP T cells in urological cancer patients may promote a Th2 profile, undermining antitumor immunity and targeting these cells could enhance type-1 immune responses.^5,34,35^ The identification of these CD4^bright+^CD8^dim+^ T cells suggests a potential phenotypic heterogeneity or unique subpopulation within the Jurkat cell population under the specific conditions of IL-6 concentration variations and associated experimental parameters.

### TNF-α and IFN-γ Levels

**Figure 5:**
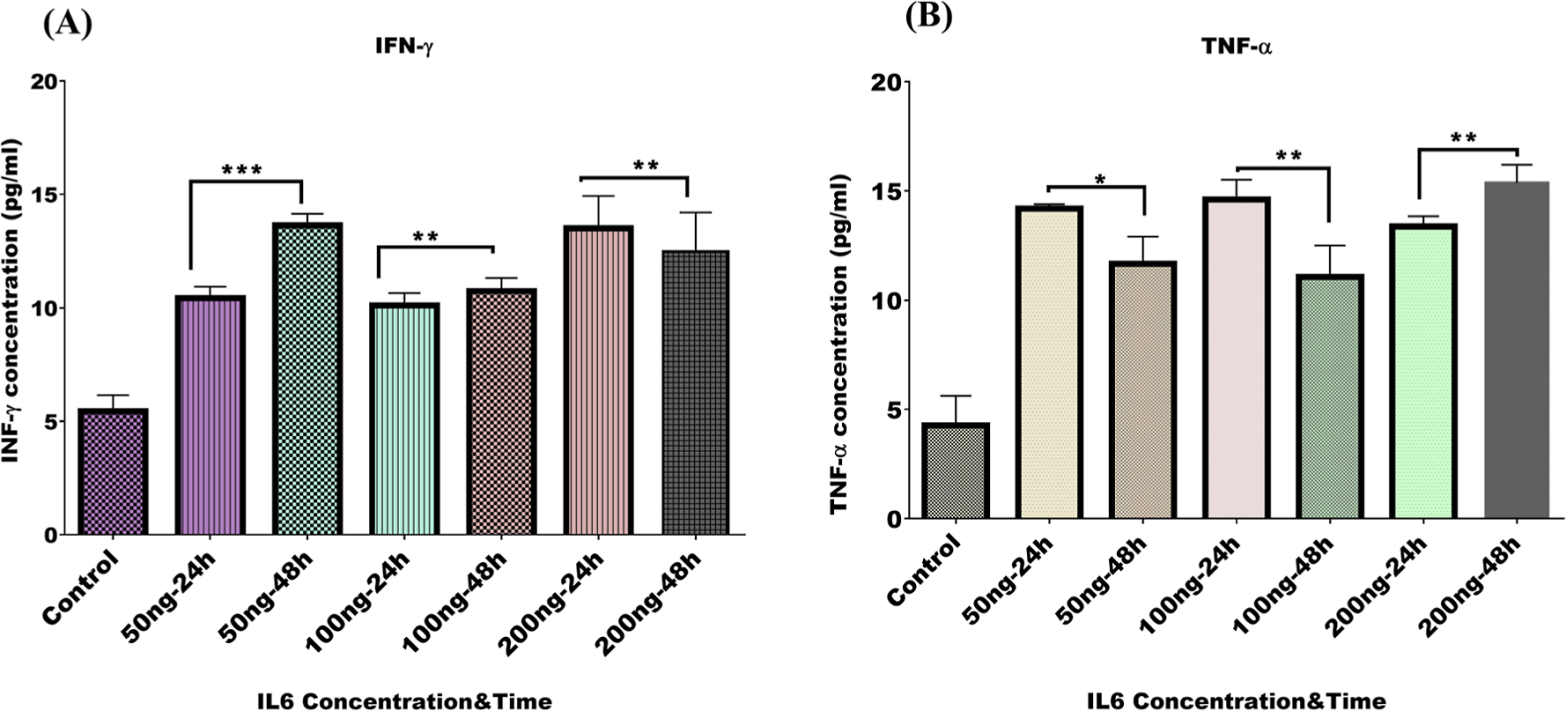
ELISA data. The bar charts show the ELISA analysis and quantification data of (A) INF-γ and (B) TNF-α protein levels after 24 and 48 h of A549/Jurkat cells cocultured on the lung tumor chip at different total IL-6 concentrations. The medium was collected from the cell outlets during passing time. All data were expressed as mean ± SEM. Statistical significance was determined by paired Student’s *t*-test. *p <0.05 was considered significant, *p <0.01, ***p <0.001.

TNF-α and IFN-γ are pleiotropic cytokines that participate in many aspects of immune regulation, especially in the TME. IFN-γ promotes the activation of the immune response and stimulates the antitumor immune response; it also prevents overactivation of the immune system and tissue damage. In chronic inflammation, TNF-α is essential in many stages of T cell development. Recent results indicated that, during full-body inflammation and the absence of infection, IL-6 induces TNF-α production by activating NF-κB.^36^ Collected medium from CCC outlets during 24 and 48 h in all experimental groups was analyzed for the level of IFN-γ and TNF-α proteins by ELISA. IFN-γ levels increased significantly from 24 to 48 h in the experimental group with a total of 50 ng IL-6 (*P* <0.0002), showing a percentage increase of approximately 30.8%. The level of IFN-γ in the 100 ng IL-6 group also showed an elevated level of IFN-γ (*P* <0.004), with a percentage increase of 24.4%. However, the level of IFN-γ gradually decreased in the total 200ng IL-6 experiment as time progressed (*P* <0.007), despite an initial increase at 24 h, indicating a possible saturation effect that led to a reduced immune response after 48 h (Figure 5A). Next, we examined the TNF-α protein level in the collected medium over 48 h. Interestingly, the level of TNF-α revealed a diminishing level during the second 24 h in both experimental groups of a total of 50 ng and 100 ng (*P* <0.041) and (*P* <0.009), with percentage decreases of 17.7% and 20.8%, respectively; however, in a total concentration of 200 ng IL-6, the concentration of TNF-α increased gradually during passing time with a percentage increase of 14.8% (*P* <0.004) (Figure 5B).

### Exhibit Exhausted Phenotype

In the TME, T cells exhibit an exhausted phenotype similar to chronic infection, marked by dysregulated expression of inhibitory receptors (CTLA-4, PD-1, TIM-3, BTLA, and LAG-3) and suppressed effector cytokines (TNF-α, IFN-γ, GzmB, and IL-2). During the passing time, variations in the concentration gradient of IL-6 influence coculture cell conditions, affecting parameters such as the rate of A549 expansion and T cell activation, possibly leading to exhaustion. Notably, levels of TNF-α and IFN-γ exhibit discernible patterns, directly impacting the T cell phase specificity within the TME. Exhausted T cells show systematic dysfunction. The impact of high IL-6 levels on IFN-γ and TNF-α expression is complex. High concentrations of IL-6 can modulate TNF-α and IFN-γ expression in immune cells, either enhancing or suppressing their production, depending on the immune context. IL-6 may promote TNF-α and IFN-γ production, amplifying inflammation, or it may induce regulatory mechanisms that reduce their levels, leading to a dampened immune response.^3,37^ Notably, TNF-α secretion is missing in the intermediate phase, and IFN-γ and granzyme B (GzmB) have vanished in the advanced phase of exhaustion.^38^ In summary, high concentrations of IL-6 could either upregulate or downregulate TNF-α and IFN-γ expression, depending on the specific immune environment and signaling pathways involved. In our investigation, PD1 expression was measured, and PD1 expression on CD4^+^ T cells increased when IL-6 was present in the system. IL-6 plays a crucial role in driving the expression of PD-L1 on cancer and immune cells, significantly contributing to T cell exhaustion, characterized by persistent expression of inhibitory receptors like PD-1, loss of effector functions, metabolic dysregulation, and epigenetic changes.^39^ Studies by Keegan et al. show that changes in plasma IL-6 levels with initial doses of PD-1 pathway inhibitors correlate with clinical responses, highlighting IL-6’s impact on PD-L1 expression.^40^ IL-6 induces PD-L1 expression in various cancers, including prostate cancer, hepatocellular carcinoma, and glioblastoma, through pathways leading to immune resistance, decreased PTPRO expression, and enhanced myeloid expression. In lung cancer, IL-6-driven PD-L1 expression is linked to multiple pathways, especially the MEK-ERK signaling pathway, enhancing inhibitory signaling and promoting an immunosuppressive microenvironment that facilitates tumor immune evasion. Clinically, targeting IL-6 signaling with IL-6 or IL-6R antibodies could reduce PD-L1 expression and T cell exhaustion, potentially improving immune responses against tumors.^41^

In a research study, 53% of 15 lung cancer cell lines expressed IL-6, indicating that overexpression may alter cytokine balance and impair antitumor immunity in lung cancer cases.^17^ IL-6 interacts with other cytokines such as IL-1, IL-23, and TGF-β in the TME, affecting CD4^+^ T cell fitness and motility. IL-1 triggers the release of proinflammatory cytokines such as IL-6 and recruits innate immune cells, initiating inflammatory responses. Tissue damage prompts the expression of IL-6, IL-10, IL-11, and IL-23, creating a self-regulating loop that resolves inflammation and promotes healing.^13^ IL-6 and IL-11 coordinate innate immune responses.^42^ IL-6 plays a pathogenic role in inflammatory diseases and acts as a double-edged sword in Th1 response. In our study, it was hypothesized that different concentrations of IL-6 would serve as both a pro- and antitumor agent in the TME. We analyzed the gradient and concentration, which are crucial factors determining immune cell activation, migration, and exhaustion phases. Here, we demonstrate that the presence of IL-6 during the coculturing of cancer cells and CD4^+^ T cells improves CD4^+^ T cells’ fitness. IL-6 levels are elevated in inflammatory conditions like COVID-19 and cancers, affecting T cell migration and immune response.^43,44^ Chronic inflammation in malignant tumors stimulates cell infiltration and cytokine production, leading to an inflammation cycle. Our investigation revealed distinct responses in A549 and Jurkat cells when exposed to varying concentrations of a chemokine gradient. With a total of 50 ng of IL-6, the expansion of A549 cells exhibits a gradual and time-dependent increase. Simultaneously, the migration of Jurkat cells also demonstrates a time-dependent pattern. In contrast, when the lung tumor chip with a total of 100 ng of IL-6 concentration was utilized, the expansion of A549 cells exhibited an exceptionally high rate. This heightened population of A549 cells potentially exerted an influence on the migration of Jurkat cells. It is conceivable that the elevated A549 population released mediators or chemokines, which, in turn, affected the migratory behavior of Jurkat cells. Simultaneously, the mobility of Jurkat cells appeared to be lower in comparison to that of the lung tumor chip with a total IL-6 concentration of 50 ng. This discrepancy in Jurkat cell mobility could be attributed to the increased presence of A549 cells and the heightened cell–cell interactions occurring on the lung tumor chip, with a total of 100 ng of IL-6. The interplay among the concentrations of IL-6, A549 cell expansion, and Jurkat cell behavior underscores the intricate dynamics of cellular interactions within this experimental setup. Interestingly, despite the cessation of A549 cell expansion in the presence of a high concentration of IL-6, the migratory behavior of Jurkat cells persisted, responding to an elevated IL-6 concentration gradient. This observation suggests a doubling of A549 cell expansion and the migratory responses of Jurkat cells under the influence of IL-6 levels. It implies that while A549 cell proliferation may be hindered, the chemotactic signals generated by the heightened IL-6 gradient continue to guide Jurkat cell migration. Diversity in cellular responses highlights the complexity of IL-6-mediated interactions and underscores the need for a nuanced understanding of the intricate signaling pathways governing cell behaviors in the experimental context. In the subsequent step, we conducted an analysis of the expression levels of various markers, including CD196, CD183, CD4, CD3, CD69, PD-1, and PDL1, on Jurkat cells within the experimental groups. Additionally, we monitored the levels of IFN-γ and TNF-α over time as part of our investigation. By assessing the expression patterns of these markers, we sought to gain insights into the molecular and immunological alterations occurring in Jurkat cells under different experimental scenarios, shedding light on the dynamic responses of these cells to the microenvironment shaped by IL-6 gradients and cell–cell interactions. The immune regulation role of PD-1 (CD279) plays a crucial role in controlling T cell activation and limiting immune-induced tissue damage by promoting self-tolerance.^44,45^ Notably, in our study, the expression of CD markers reveals early activation in T cells, as evidenced by the upregulation of CD69. This suggests a dynamic interplay among the chemokine gradient, IL-6 concentration, and the distinct cellular behaviors exhibited by A549 and Jurkat cells, highlighting the intricate nature of these responses in the experimental setting. The observation suggests that IL-6 signaling reaches a saturation point at 50 ng/mL, beyond which increasing the concentration does not further enhance the immune response. This threshold effect is common in signaling pathways, where receptors become fully activated and additional ligands do not result in a stronger signal. This finding is significant for understanding the efficacy of IL-6 in our experiments, as it indicates that 50 ng/mL is the optimal concentration for activating the CD markers. Concurrently, the increased levels of IFN-γ and TNF-α further emphasize the multifaceted impact of IL-6, potentially steering T cells toward an inflammatory response characterized by heightened cytokine production. In our study, we observed that PD-L1 expression on experimental T cells was higher compared with the control group. Previous research has well established that proinflammatory cytokines, such as IFN-γ, can induce PD-L1 expression.^46^

PD1/PD-L1 inhibitors unlock antitumor activity, reducing phagocytic activity. High PD-L1 expression in TMEs is due to oncogenic signaling and inflammatory factors. Blocking PD-1 restores T cell function, improving pathogen control and tumor eradication.^47^ Prolonged antigen stimulation and inflammation lead to the loss of effector functions.^48^ A significant observation was the pronounced expression of PDL1 by A549 cells. This robust expression of PDL1 by the A549 cell population holds noteworthy implications for immune regulation. The heightened expression of PDL1 by A549 cells suggests a potential immunomodulatory strategy that is employed by these cells. This could influence the dynamics of T cell interactions within the microenvironment. Therapeutically targeting IL-6 or its signaling pathway in cancer has several clinical implications for modulating the immune response. It can inhibit tumor growth and overcome resistance to conventional therapies by reducing IL-6’s protective effects on cancer cells. Combining IL-6 inhibitors with chemotherapy has shown promising results, although its combination with immune checkpoint inhibitors is controversial due to potential reductions in PD-1 and PDL1 expression. Blocking IL-6 trans-signaling shows potential in specific cancers like hepatocellular carcinoma and colorectal cancer, with drugs like olamkicept (soluble gp130Fc) representing promising targeted therapies.^49^ However, IL-6 inhibitors can also increase infection risks, necessitating balanced risk management. Understanding IL-6’s role in individual cancers could lead to personalized treatment strategies, enhancing the effectiveness of cancer therapies. By examining a range of concentrations and gradients, researchers can delineate how varying IL-6 levels influence T cell migration and cancer cell proliferation, providing insights into dose-dependent effects and gradient dynamics. This helps identify critical thresholds and optimize therapeutic strategies for targeting cytokine-driven cancer progression.

## Conclusions

In summary, in a 3D microenvironment with a constant-flow concentration of IL-6, cell-embedded GelMA hydrogel was placed in CCAs, and due to the design, each chamber experienced a different concentration gradient of IL-6. The cell–cell interaction and efficacy of the chip were observed and analyzed via our flow-based tumor lab chip. We demonstrated the potential value of our novel designed 3D tumor-on-a-chip device in TME research by exploring the effect of different gradients of IL-6 and the dual impact of IL-6 on T cells and lung cancer cells in a TME simulation.

## Supporting Information Available

Lung tumor chip illustration; 5% GelMA hydrogel pore size evolution over time; hydrogel stability, cell viability, and swelling rate over time; cell viability of Jurkat and A549 cells encapsulated in 5% (w/v) GelMA; effects of different IL-6 concentrations on Jurkat T cells’ and A549 cells’ viability over 48 h on a plate; effects of diluted 50, 100, and 200 ng pumped IL-6 into the lung TME chip on cocultured cancer cells and immune cells; CD4/CD8/CD69/CD183/CD196/PD1/PDL1 marker expression on the Jurkat cell surface monocultured in the flask; flow cytometry data; image processing and A549 cell counting workflow; fluorescence microscope parameters and filters; cell counting data for A549 and Jurkat cells at varying IL-6 concentrations in biochip; raw data of cell viability assessment on biochip using LIVE/DEAD assays; raw data regarding the cell viability in 5% GelMA assessed over three days in the 96-well plate using the LIVE/DEAD assay; and key experimental details for image analysis (PDF)
Biochip gradient stimulator structure (MP4)
Jurkat cells were cultured after 16 h in RPMI + 10% FBS + IL-6. CellTracker Red CMTPX dye was used (MP4)

## Abbreviations

IL-6: interleukin 6
TME: tumor microenvironment
TAM: tumor-associated macrophage
MDSC: myeloid-derived suppressor cell
Treg: regulatory T CD4^+^
TNFα: tumor necrosis factor-α
TGF-β: transforming growth factor-β
IL-1: interleukin-1
IFN-γ: interferon gamma
GPCR: G-protein-coupled receptor
PDMS: polydimethylsiloxane
CCAs: cell culture areas
CCCs: cell collection chambers
GelMA: gelatin methacrylate
SEM: scanning electron microscopy
EMTs: epithelial-mesenchymal transitions
NSCLC: nonsmall cell lung cancer
DP T cells: double positive T cells
PD-1: programed cell death protein 1
PDL1: programed death-ligand 1
HRFEGSEM: high-resolution thermal field emission scanning electron microscopy
PBS: phosphate-buffered saline
DPBS: Dulbecco’s phosphate-buffered saline
MA: methacrylic anhydride;
DMEM: Dulbecco’s modified Eagle’s medium
FBS: fetal bovine serum
CMFDA: 5-chloromethyl fluorescein diacetate
