# Supplementary material for: Investigating T Cell Immune Dynamics and IL-6’s Duality in a Microfluidic Lung Tumor Model
Source: ACS Appl Mater Interfaces. 2024 Oct 29;17(3):4354–67. doi: 10.1021/acsami.4c09065 (PMC11758792; doi:10.1021/acsami.4c09065)
Supplement: Supplementary file 1 — am4c09065_si_001.pdf [file am4c09065_si_001.pdf]

Supporting Information

Investigating T Cell Immune Dynamics and IL-6's Duality in a Microfluidic Lung-Tumor Model

Parvaneh Sardarabadi[1], Kang-Yun Lee[2,3,4], Wei-Lun Sun[5], Amir Asri Kojabad[6], Cheng-Hsien Liu[1,7,8]*

[1]Institute of Nanoengineering and Microsystems, National Tsing Hua University, Hsinchu, 30044, Taiwan, R.O.C.

[2]Division of Pulmonary Medicine, Department of Internal Medicine, Shuang Ho Hospital, Taipei Medical University, New Taipei City 235, Taiwan, R.O.C.

[3]Division of Pulmonary Medicine, Department of Internal Medicine, School of Medicine, College of Medicine, Taipei Medical University, Taipei 110, Taiwan, R.O.C.

[4]TMU Research Center for Thoracic Medicine, Taipei Medical University, Taipei 110, Taiwan, R.O.C.

[5]Pythia Biotech LTD., New Taipei City 23561, Taiwan, R.O.C.

[6]Department of Hematology, School of Allied Medical Sciences, Iran University of Medical Sciences, Tehran 14535, Iran

[7]Department of Power Mechanical Engineering, National Tsing Hua University, Hsinchu, 30044, Taiwan, R.O.C.

[8]College of Semiconductor Research, National Tsing Hua University, Hsinchu, 30044, Taiwan, R.O.C

* Corresponding author : Cheng-Hsien Liu ; E-mail : liuch@pme.nthu.edu.tw; Tel: +886-3-5742496

This Supplementary file includes:

Fig. S1. Lung tumor chip illustration
Fig. S2. 5% Gelatin Methacrylate (GelMA) hydrogel pore size evolution over time
Fig. S3. Hydrogel stability, cell viability, and swelling rate over time
Fig. S4. Cell viability of Jurkat &A549 cells encapsulated in 5% (w/v) GelMA
Fig. S5. The effects of different IL-6 concentrations on Jurkat T cells and A549 cells' viability over 48 hours on a plate
Fig. S6. Effects of diluted 50ng, 100ng, 200ng pumped IL-6 into the lung-tumor microenvironment chip on cancer cells and immune cells co-cultured

Fig. S7. The charts display CD4 /CD8 /CD69 / CD183/ CD196 /PD1 / PDL1 markers expression on Jurkat cells surface were mono-cultured in the flask.
Fig. S8. Flow cytometry data
Fig. S9. Image processing and A549 cell counting workflow

Table S1. Florescent Microscope parameters and filters
Table S2. Flow cytometry data
Table S3. Cell counting data for A549 and Jurkat cells at varying IL-6 concentrations in biochip
Table S4. Raw data of cell viability assessment on biochip using Live/Dead assays
Table S5. Raw data regarding the cell viability in 5% GelMA was assessed over three days in the 96-well plate using LIVE/DEAD assay.
Table S6. Key experimental details for image analysis

Movie S1. Biochip gradient stimulator structure
Movie S2. Jurkat cells after 16 hours in RPMI+10%FBS+IL-6. Red (CellTracker™ Red CMTPX Dye)

Figure S1

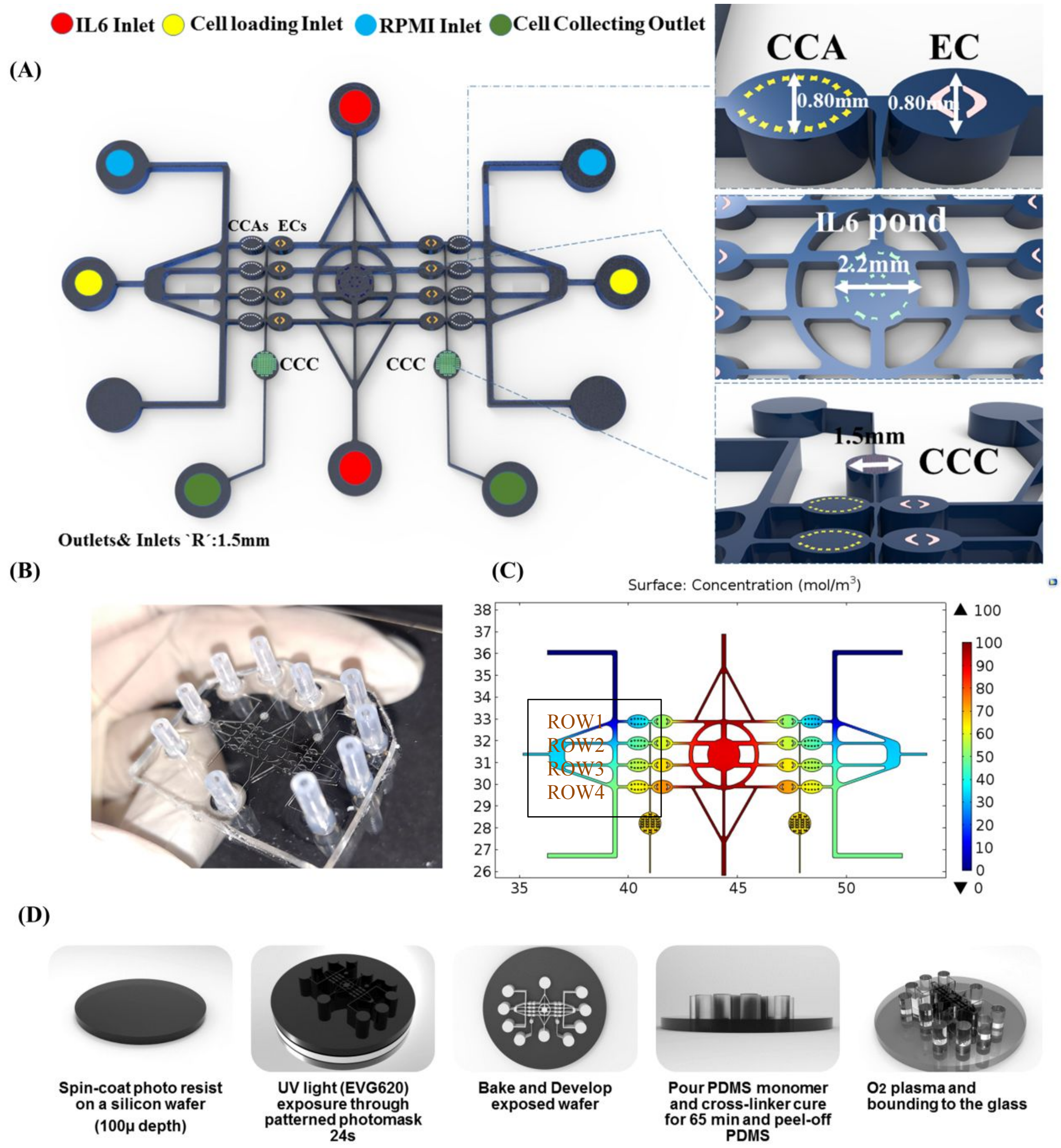

Figure S1. Lung tumor chip illustration. (A) The full chip principle and dimensions are presented here. The width of CCAs is 0.8 mm, and the diameter of CCCs is 1.5mm. (B) Fabricated lung-tumor chip (C) Modeling and simulation of gradient concentrations with COMSOL show different concentration gradients of IL-6 per ROW (ROW1-ROW4) between CCAs and ECs. (D) During the fabrication process, the photoresist (SU8) is poured on the wafer and then spun coated to create 100μm depths of photoresist. The wafer was soft-baked for 24 seconds and then exposed to the EVG620 machine for lithography. The wafer hard-baking process and SU8 developer processing were used to prepare the proper design for the wafer. A lung tumor microenvironment chip is created using PDMS and bound to the glass in the final step.

Figure S2

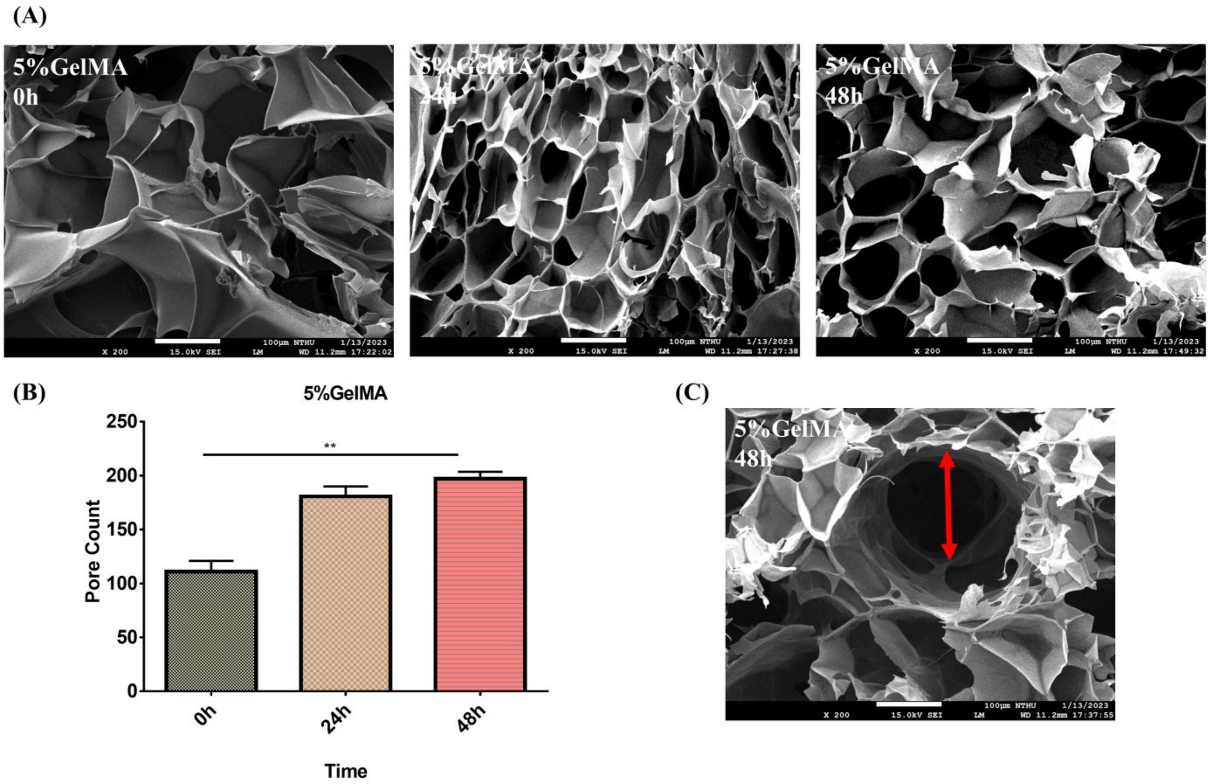

Figure S2. 5% Gelatin Methacrylate (GelMA) hydrogel pore size evolution over time. (A) Comparison of hydrogel pore size alterations over 0, 24, and 48 hours, captured using a High-Resolution Thermal Field Emission Scanning Electron Microscope. scale bar 100µm, 15 kV, and WD = 11.2 mm, sputtering times the 90s and current 20Ma. (B) GelMA quantitative analysis of pores during 0-24-48 hours One-Way-ANOVA, statistically significant ($P < 0.05$) (n=3). $**P < 0.007$. (C) A large Pore size after 48h.

Figure S3

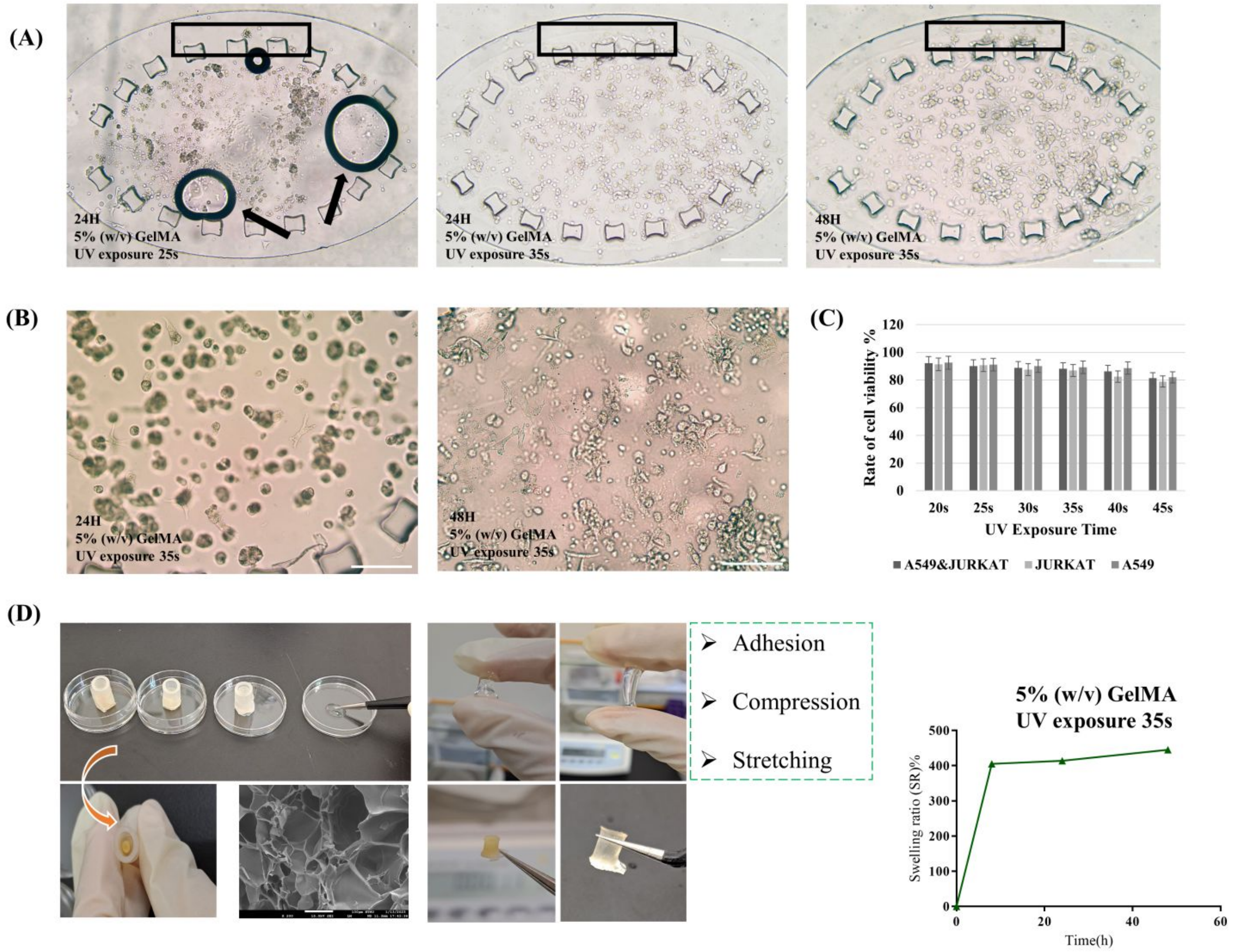

Figure S3. Hydrogel stability, cell viability, and swelling rate over time. (A) Stability tests showed structural integrity under constant flow for 35 seconds. Squares show the margin of hydrogels during passing time in the biochip. Black arrows show bubbles. (B) and (C)show cell viability experiments during the time (24h) and (48h) and the results confirmed ~80% cell viability after 35 seconds of incubation. (D) Adhesion, compression, and stretching were examined, and the swelling rate of GelMA hydrogels was measured over a 48-hour period. The scale bar is 100 µm.

Figure S4

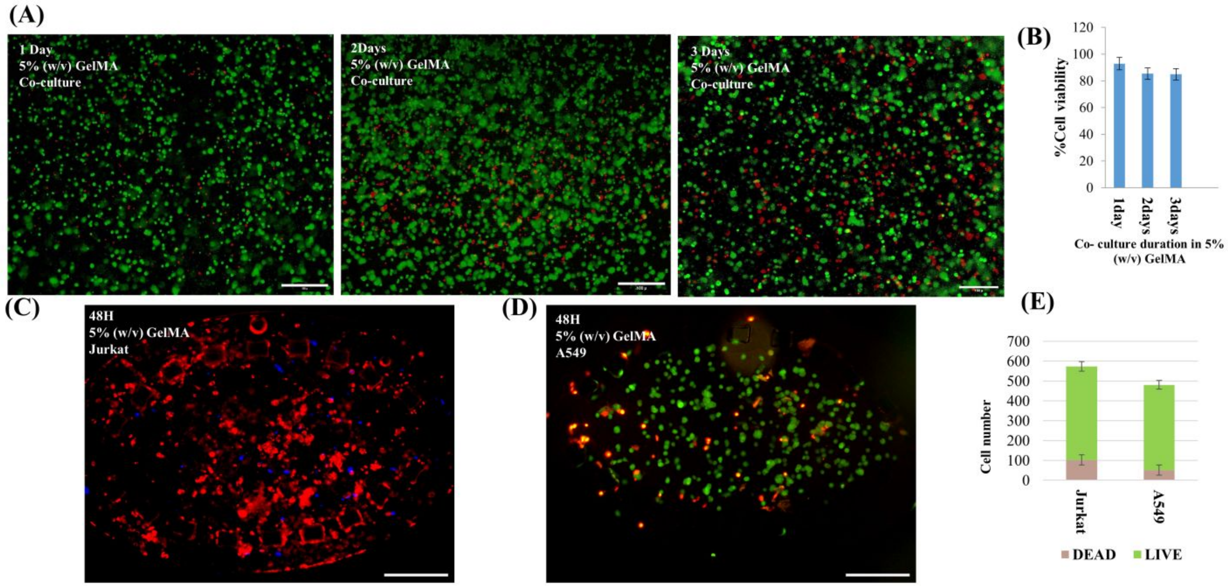

Figure S4. Cell viability of Jurkat and A549 cells encapsulated in 5% (w/v) GelMA in standard medium (RPMI + 10% FBS). (A) Cell viability in 5% GelMA, assessed using the LIVE/DEAD Thermo Fisher Scientific assay on Day 1, Day 2, and Day 3 (red: dead, green: live) in a 96-well plate with the standard medium. (B) Cell viability was ≥ 84%. (C) Jurkat cell viability on the biochip assessed with the Live/Dead™ Cell Viability Assay Kit (blue: dead, red: live). (D) A549 cell viability assessed with the LIVE/DEAD Thermo Fisher Scientific assay (red: dead, green: live) after 48 hours. (E) Cells cultured in standard medium showed ≤ 82% and ≥ 88% viability, respectively, after 48 hours (n = 5). * indicates p-value < 0.05. Scale bar: 200 μm.

Figure S5

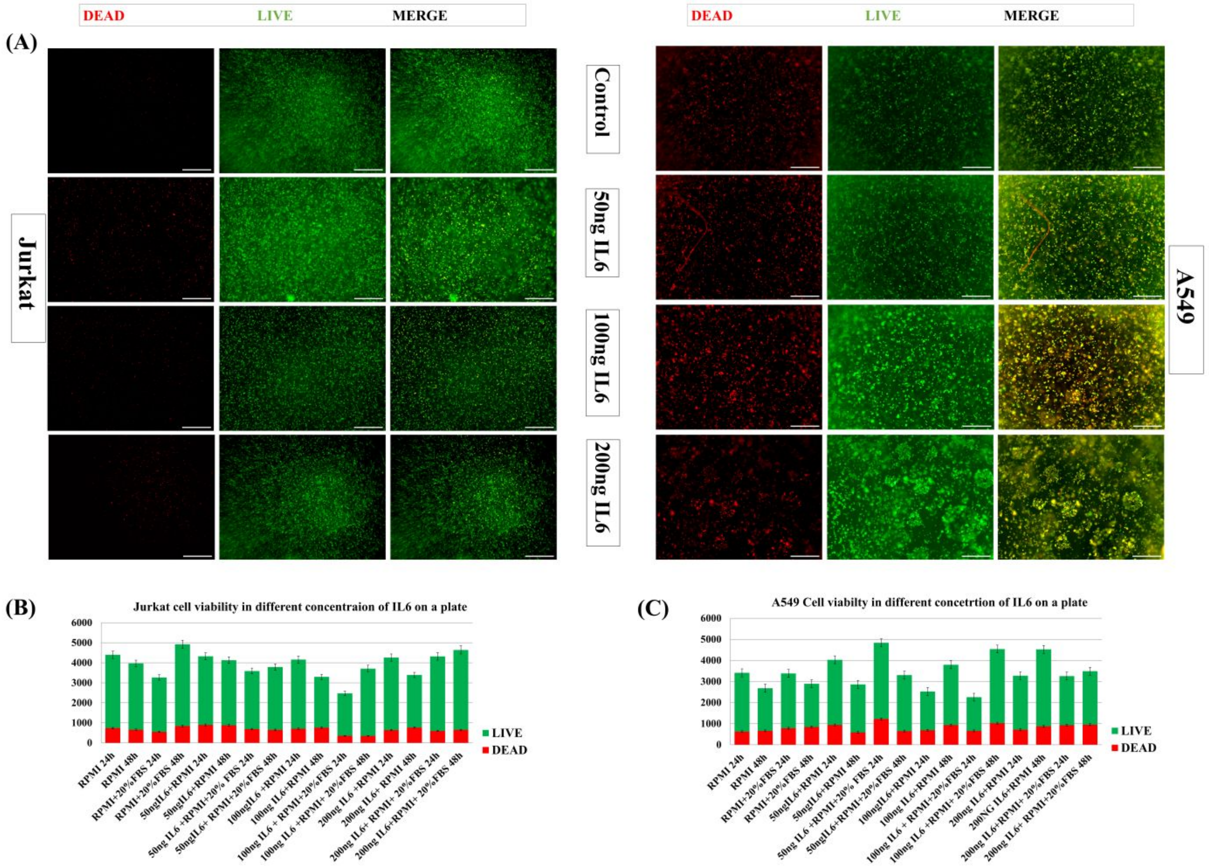

Figure S5. The effects of different IL-6 concentrations on Jurkat T cells and A549 cells' viability over 48 hours on a plate. Jurkat cells and A549 cells were cultured in the presence of the standard medium (RPMI + 10% FBS) as well as in the medium containing 20% FBS (controls). (A) The survival status of Jurkat and A549 cells for 48 hours was observed using a fluorescence microscope with LIVE/ DEAD (green/ red) assay (n = 5). (B) Jurkat cell viability (green) and mortality (red) rate. In 50 ng/mL IL-6 + RPMI + 20% FBS, viability was 85.7% at 24 hours ($p \leq 0.01$) and 82.6% at 48 hours ($p \leq 0.03$). In 100 ng/mL IL-6 + RPMI + 20% FBS, viability was 86.5% at 24 hours ($p \leq 0.02$) and 83.3% at 48 hours ($p \leq 0.03$). In 200 ng/mL IL-6 + RPMI + 20% FBS, viability was 86.8% at 24 hours ($p \leq 0.005$) and 84% at 48 hours ($p \leq 0.01$). (C) A549 viability (green) and mortality (red) rate. 50 ng/mL IL-6 + RPMI + 20% FBS: viability was 80% at 24 hours ($p \leq 0.005$) and 78% at 48 hours ($p \leq 0.01$). 100 ng/mL IL-6 + RPMI+ 20% FBS: viability was 79% at 24 hours ($p \leq 0.01$) and 77% at 48 hours ($p \leq 0.03$), 200 ng/mL IL-6 + RPMI + 20% FBS: viability was 80% at 24 hours ($p \leq 0.005$) and 78% at 48 hours ($p \leq 0.01$). The data represent the mean ± SEM. * indicates p-value < 0.05. Scale bar 200μm.

Figure S6

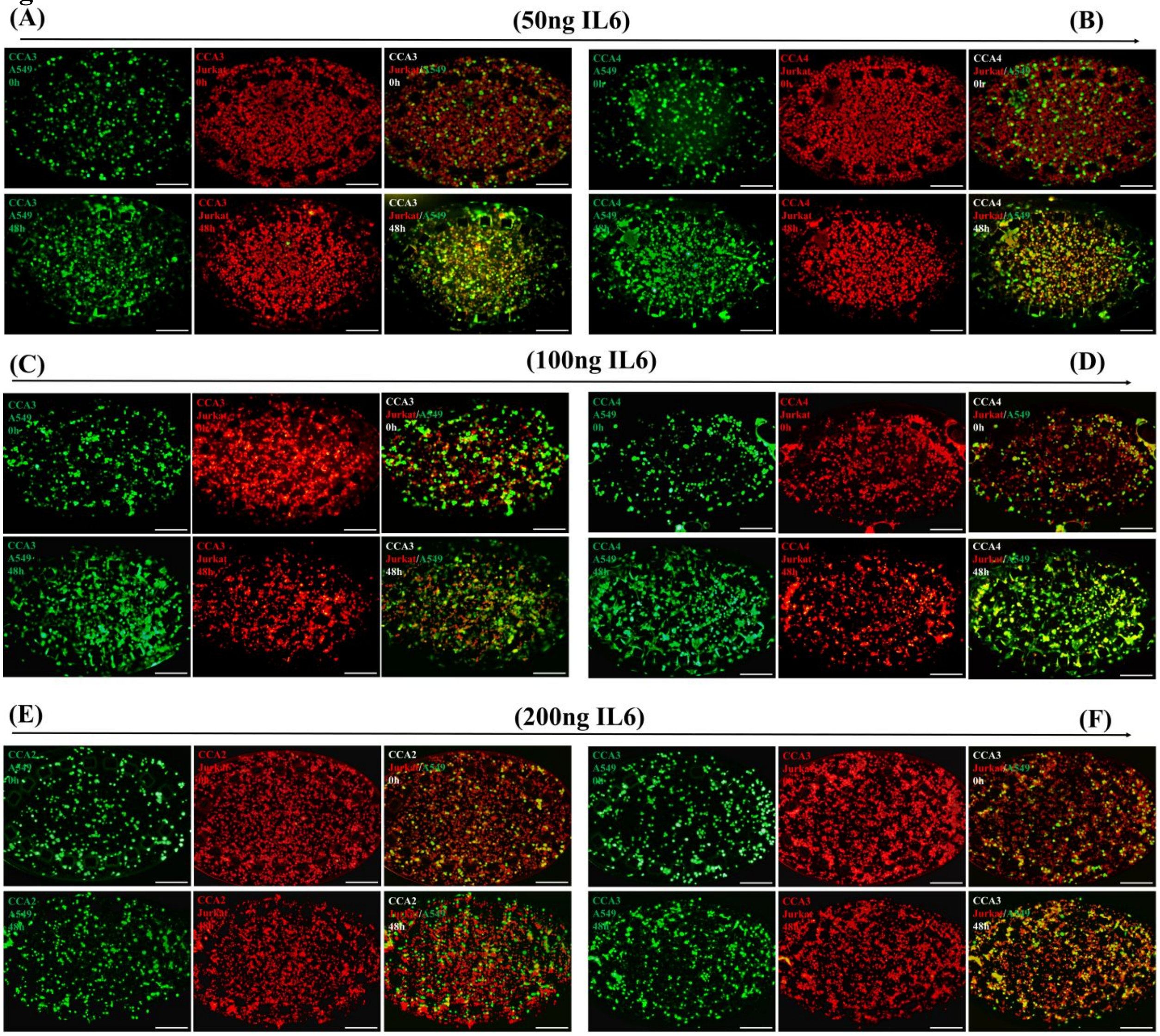

Figure S6. Effects of diluted 50 ng/mL, 100 ng/mL, and 200 ng/mL pumped IL-6 into the lung-tumor microenvironment chip on cancer cells and immune cells co-cultured. (A) CCA3, co-culture of A549 (green)/ Jurkat (red), observation in 0h-48h, (B) CCA4, co-culture of A549 (green)/ Jurkat (red), observation in 0-48 hours. Following chambers exposed to 100 ng IL-6, (C) CCA3, co-culture of A549 (green)/ Jurkat (red) and (D) CCA4, co-culture of A549 (green)/ Jurkat (red), monitoring in 0-48 hours. (E) and (F) CCA2 and CCA3 in present of 200 ng. The scale bar is 200 μm.

Figure S7

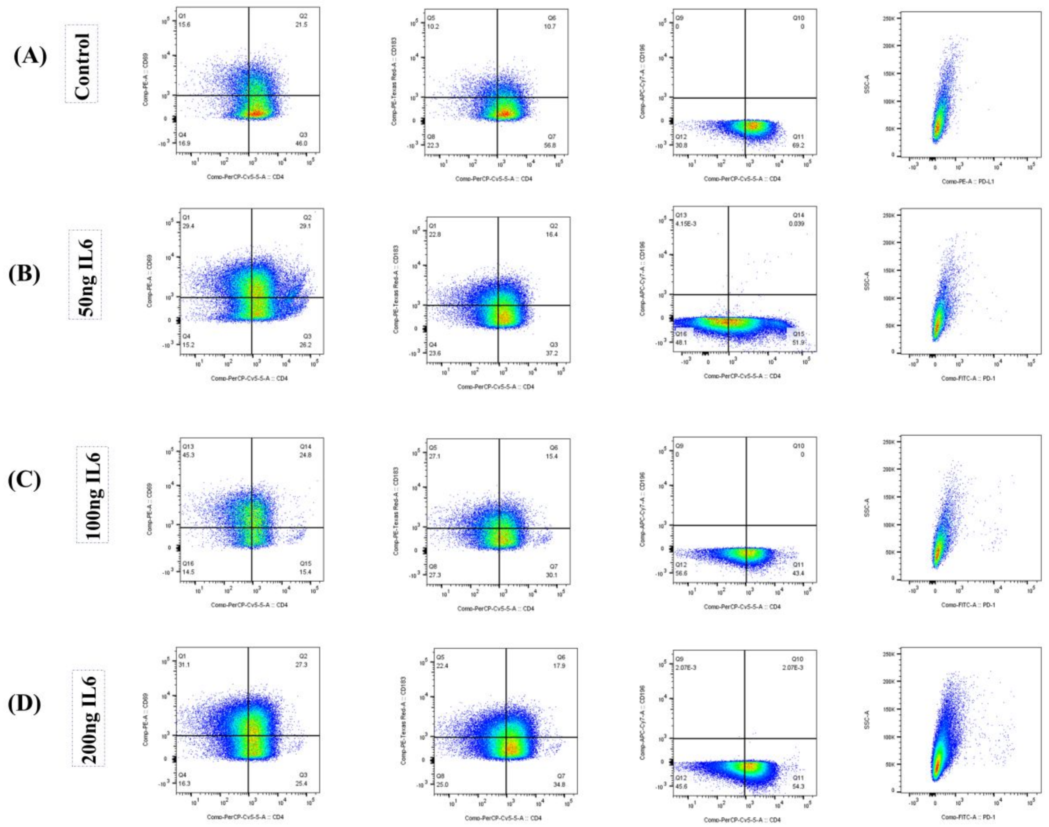

(E)

CD markers	Control	50ng IL6	100ng IL6	200ng IL6
CD69+CD4+	21.5%	29.1%	24.9%	27.3%
CD183+CD4+	10.7%	16.4%	15.4%	17.9%
CD8+CD4+	0	0	0	0
CD196+CD4+	0	0	0	0
PD1+PDL1+	1.22%	1.94%	2.84%	1.75%
PD1+	0.87%	1.93%	2.04%	1.53%

Figure S7. The charts display CD4 /CD8 /CD69 / CD183/ CD196 /PD1 /PDL1 markers expression on Jurkat cells surface were mono-cultured in the flask. (A) A control group was cultured for 48 hours in RPMI +20 % FBS without the presence of IL-6, (B) experiment group cultured for 48 hours with 50 ng/mL IL-6+ RPMI +20 % FBS (C) experiment cultured for 48 hours with 100 ng/mL IL-6+ RPMI +20 % FBS (D) experiment cultured for 48 hours with 200 ng/mL IL-6+ RPMI +20 % FBS, analyzed by FLOWJO. (E) The table presents differences in the expression of CD4 /CD8 /CD69 /CD183 /CD196 /PD1 /PDL1 between the control and experiment groups.

Figure S8

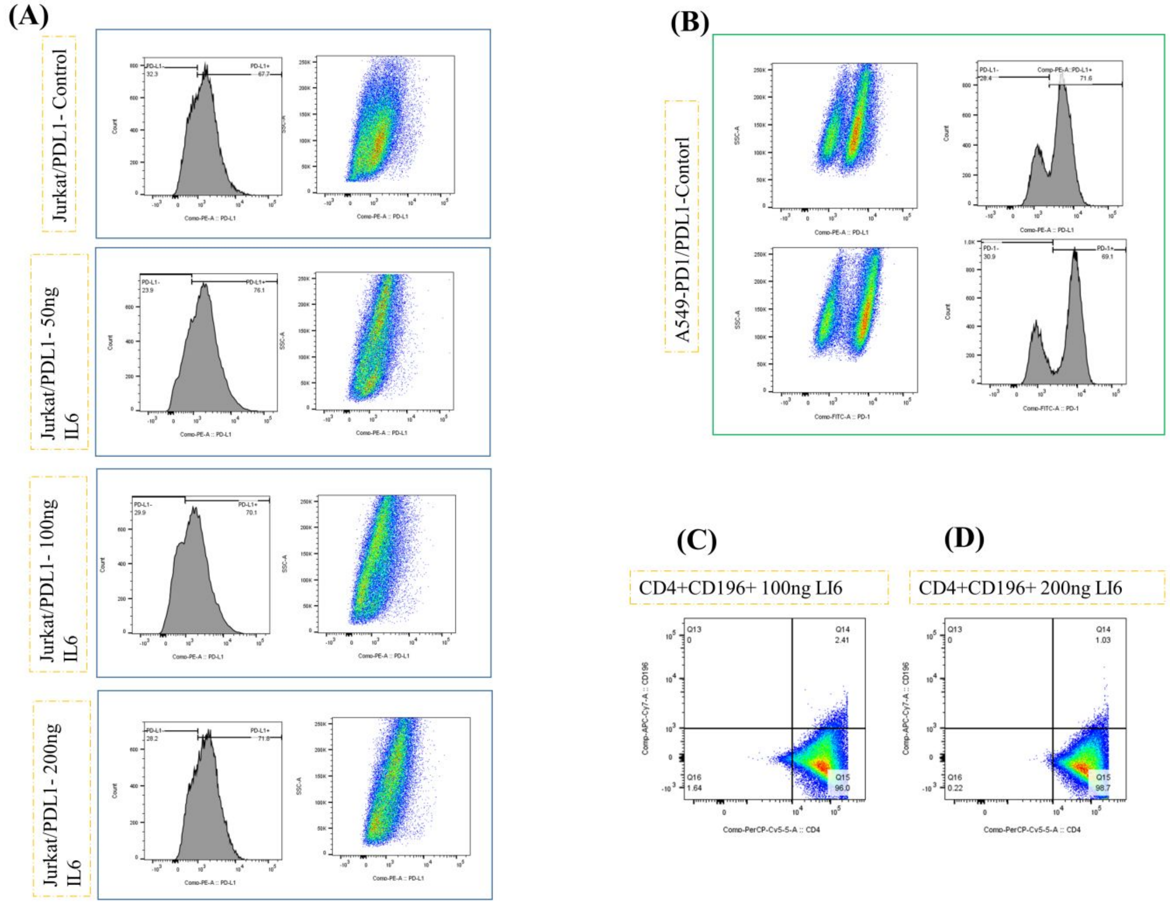

Figure S8. Flow cytometry data. (A) PDL1 marker expression on the surface of Jurkat cells co-cultured in the biochip. (B Expression of PD1/PDL1 on A549 control group cultured for 48 hours in RPMI + 20 % FBS without IL-6. (C) $CD4^{+}CD196^{+}$ population in the presence of 100 ng/mL IL-6 (D) $CD4^{+}CD196^{+}$ population in the presence of 200 ng/mL IL-6. Data were analyzed using FlowJo.

Figure S9

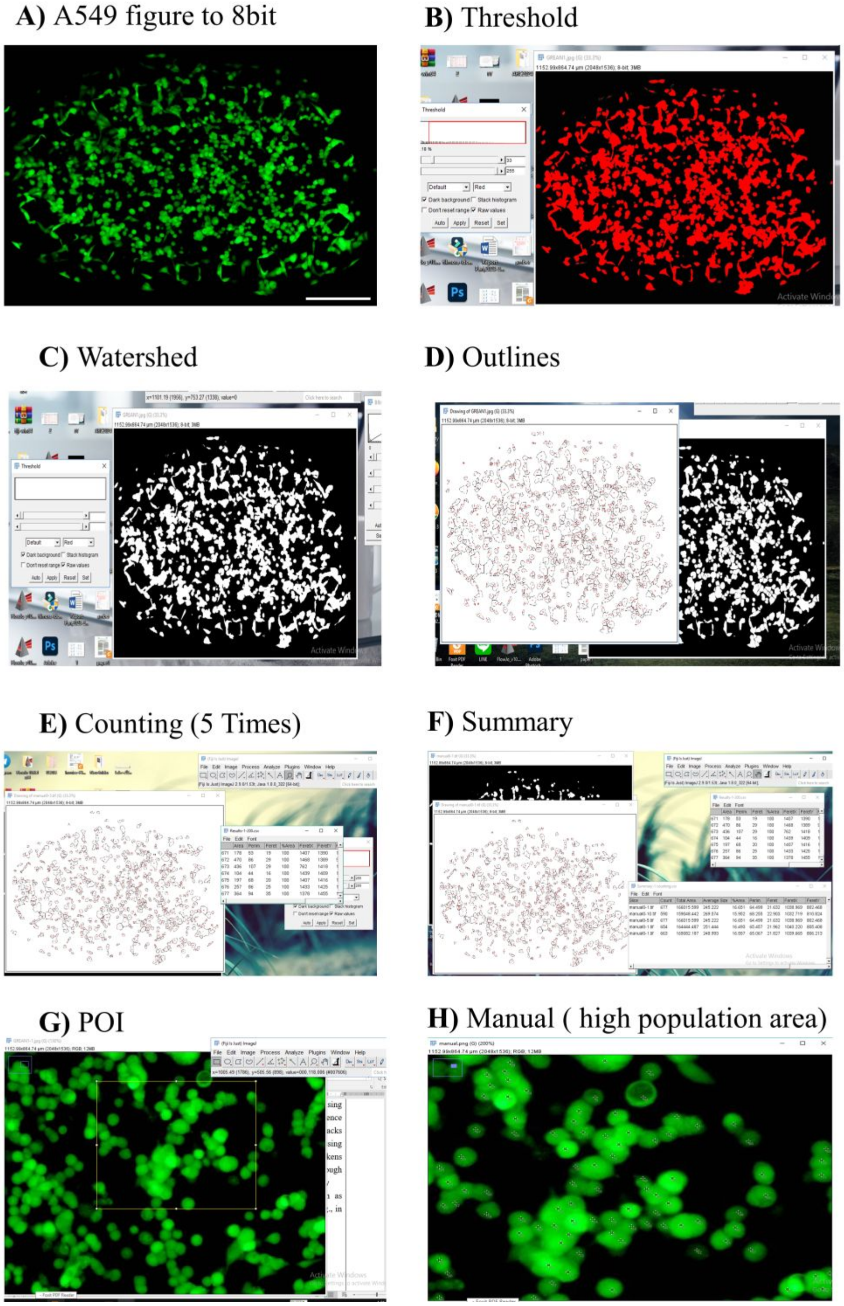

Figure S9. Image processing and A549 cell counting workflow. (A-F) This figure outlines the key steps involved in the ImageJ cell counting process, from image conversion and thresholding to (G-H) manual counting and summary generation.

Table S1. Florescent Microscope parameters and filters

**Parameter**	**Details**
Microscope	Olympus BX 51 Fluorescent Microscope
Filter	Nomarski filter for differential interference contrast (DIC) microscopy
Illumination Source	100 W quartz halogen light source
Camera	FPC-3L310OIII (Forever Plus, Taipei, Taiwan)
Software	Forever Plus software (Forever Plus Corp, Taipei, Taiwan)
Objectives	Olympus LMPlanFI 10×/0.25, Olympus MPLFLN 5X, Olympus LMPLFLN 20X
Image Resolution	3.5401 pixels per µm
Pixel Size	0.2825 x 0.2825 μm^2
Pixel Depth	32 bits (RGB)
Magnification	0.33x (Fiji ImageJ)
Fields of View	Multiple fields to ensure comprehensive data
Replicates	5 replicates, 3 distinct areas per sample
**Exposure Times**	
Live & Dead Fluorescent Green Labeled Sample	489.06 ~ 506.071ms
Live & Dead Fluorescent Red Labeled Sample	89.291 ~ 107.165ms
Cell Tracker Fluorescent Green Labeled Sample	90.616 ~ 130.4ms
Cell Tracker Fluorescent Red Labeled Sample	120.4 ~ 180.4ms
Cell Tracker Fluorescent Blue Labeled Sample	226.75 ~ 344.6ms
Exposure Time for Bright Field Figures	~ 9.674ms
**Filter information**	
U-MWU2	Excitation: 330-385 nm, Emission: 420 nm, Application: UV light, Blue dye
U-MWB2	Excitation: 460-490 nm, Emission: 520 nm, Application: Live & dead (simultaneous detection of green and red light)
U-MWG2	Excitation: 510-550 nm, Emission: 590 nm, Application: CMXPT, live & dead
U-MNIBA3	Excitation: 470-495 nm, Emission: 510-550 nm, Application: CMFDA, live & dead

Table S2. Flow cytometry data

To evaluate the immune phenotypic characteristics of Jurkat cells, monoclonal antibodies (anti-human) were used.

Fluorochrome	Antigen	Brand	Cat No	clone	CTL IgG
APC-R700	CD3	BD	565119	UCHT1	Mouse IgG1, κ
PerCP/Cyanine5.5	CD4	Biolegend	317428	OKT4	Mouse IgG2b, κ
BUV395	CD8	BD	563795	RPA-T8	Mouse IgG1, κ
PE	CD69	Biolegend	310906	FN50	Mouse IgG1, κ
PE/Cy5	CD183 (CXCR3)	Biolegend	353756	G025H7	Mouse IgG1, κ
APC/Fire™ 750	CD196 (CCR6)	Biolegend	353443	G034E3	Mouse IgG2b, κ
FITC	PD-1(CD279)	BD	557860	MIH4	Mouse IgG1, κ
PE	PD-L1 (B7-H1,CD274)	Biolegend	374512	MIH3	Mouse IgG1, κ

Table S3. Cell counting data for A549 and Jurkat cells at varying IL-6 concentrations

50ng CCA1 0H Jurkat	50ng CCA1 24H Jurkat	50ng CCA1 48H Jurkat	50ng CCA2 0H Jurkat	50ng CCA2 24H Jurkat	50ng CCA2 48H Jurkat	50ng CCA3 0H Jurkat	50ng CCA3 24H Jurkat	50ng CCA3 48H Jurkat	50ng CCA4 0H Jurkat	50ng CCA4 24H Jurkat	50ng CCA4 48H Jurkat
*1743.	1221.	*1057.	*1471.	1170.	*1160.	*1658.	1204.	*1052.	*2008.	1173.	*881.
*1526.	966.	*907.	*1540.	1000.	*960.	*1383.	993.	*959.	*1462.	1075.	*941.
*1559.	915.	*787.	*1367.	1026.	*993.	*1511.	1050.	*1005.	*1257.	924.	*827.
*1650.	1202.	*1060.	*1399.	998.	*903.	*1605.	1198.	*1031.	*2020.	1185.	*943.
*1601.	1036.	*919.	*1497.	996.	*932.	*1341.	1009.	*965.	*1487.	1110.	*995.

50ng CCA1 0H A549	50ng CCA1 24H A549	50ng CCA1 48H A549	50ng CCA2 0H A549	50ng CCA2 24H A549	50ng CCA2 48H A549	50ng CCA3 0H A549	50ng CCA3 24H A549	50ng CCA3 48H A549	50ng CCA4 0H A549	50ng CCA4 24H A549	50ng CCA4 48H A549
*610.	634.	*1042.	*500.	772.	*1022.	*669.	791.	*1408.	*545.	684.	*713.
*710.	774.	*942.	*548.	699.	*1070.	*462.	643.	*805.	*477.	491.	*928.
*406.	445.	*750.	*306.	451.	*850.	*474.	498.	*782.	*249.	332.	*722.
*668.	817.	*1260.	*575.	635.	*1086.	*849.	1040.	*1213.	*312.	405.	*801.
*594.	683.	*1203.	*593.	789.	*998.	*509.	605.	*913.	*334.	475.	*699.

100ng CCA1 0H Jurkat	100ng CCA1 24H Jurkat	100ng CCA1 48H Jurkat	100ng CCA2 0H Jurkat	100ng CCA2 24H Jurkat	100ng CCA2 48H Jurkat	100ng CCA3 0H Jurkat	100ng CCA3 24H Jurkat	100ng CCA3 48H Jurkat	100ng CCA4 0H Jurkat	100ng CCA4 24H Jurkat	100ng CCA4 48H Jurkat
1162.	*1166.	*1074.	1060.	*1144.	*972.	919.	*998.	*787.	860.	*907.	*882.
1310.	*1442.	*1166.	1186.	*1212.	*1004.	893.	*1107.	*1078.	725.	*988.	*886.
850.	*1070.	*885.	1044.	*1194.	*938.	1118.	*1198.	*872.	1102.	*966.	*882.
1044.	*1239.	*1088.	1147.	*1251.	*1067.	1109.	*1233.	*808.	998.	*910.	*775.
1469.	*1703.	*1208.	1558.	*1737.	*1020.	1007.	*1112.	*1018.	833.	*1312.	*1169.

100ng CCA1 0H A549	100ng CCA1 24H A549	100ng CCA1 48H A549	100ng CCA2 0H A549	100ng CCA2 24H A549	100ng CCA2 48H A549	100ng CCA3 0H A549	100ng CCA3 24H A549	100ng CCA3 48H A549	100ng CCA4 0H A549	100ng CCA4 24H A549	100ng CCA4 48H A549
*644.	875.	*1536.	*614.	1871.	*2819.	*919.	1209.	*2093.	*605.	1141.	*1432.
*705.	900.	*1753.	*655.	1316.	*1816.	*893.	1107.	*1878.	*388.	981.	*1345.
*877.	1136.	*2272.	*716.	1786.	*2813.	*1115.	1286.	*1969.	*576.	1172.	*1387.
*674.	891.	*2154.	*615.	1666.	*3005.	*1114.	1197.	*2089.	*432.	809.	*1306.
*860.	1119.	*1669.	*807.	1179.	*1408.	*505.	887.	*1340.	*491.	1102.	*1282.

200ng CCA1 0H Jurkat	200ng CCA1 24H Jurkat	200ng CCA1 48H Jurkat	200ng CCA2 0H Jurkat	200ng CCA2 24H Jurkat	200ng CCA2 48H Jurkat	200ng CCA3 0H Jurkat	200ng CCA3 24H Jurkat	200ng CCA3 48H Jurkat	200ng CCA4 0H Jurkat	200ng CCA4 24H Jurkat	200ng CCA4 48H Jurkat
*1400.	1029.	*941.	*1187.	1053.	*1027.	*965.	939.	*920.	*1277.	1177.	*1070.
*1838.	1649.	*1501.	*2021.	1761.	*1691.	*1824.	1407.	*1394.	*1535.	1189.	*1105.
*1631.	1443.	*1411.	*1550.	1424.	*1401.	*1688.	1442.	*1421.	*1562.	1475.	*1425.
*1232.	1011.	*968.	*1449.	1177.	*958.	*1256.	1119.	*1082.	*1669.	1538.	*1244.
*1395.	1114.	*1016.	*1136.	1066.	*1044.	*1142.	1069.	*990.	*1070.	869.	*801.

200ng CCA1 0H A549	200ng CCA1 24H A549	200ng CCA1 48H A549	200ng CCA2 0H A549	200ng CCA2 24H A549	200ng CCA2 48H A549	200ng CCA3 0H A549	200ng CCA3 24H A549	200ng CCA3 48H A549	200ng CCA4 0H A549	200ng CCA4 24H A549	200ng CCA4 48H A549
*581.	786.	*995.	*407.	821.	*976.	*454.	676.	*829.	*406.	500.	*614.
*461.	558.	*611.	*499.	715.	*896.	*457.	593.	*699.	*429.	585.	*602.
*825.	900.	*984.	*907.	921.	*967.	*838.	912.	*993.	*595.	601.	*662.
*643.	787.	*1105.	*513.	593.	*623.	*538.	569.	*866.	*383.	401.	*449.
*639.	725.	*865.	*503.	515.	*580.	*568.	692.	*920.	*362.	483.	*545.

Table S4. Raw data of cell viability assessment on biochip using Live/Dead assays

Jurkat Cell		A549	
DEAD	LIVE	DEAD	LIVE
69	326	68	381
86	431	45	480
122	504	47	476
136	519	56	401
98	577	41	409

Table S5. Raw data regarding to the cell viability in 5% GelMA, assessed over three days in the 96-well plate using LIVE/DEAD assay

Day	Live Cells	Dead Cells
Day1	3242	234
	3264	256
	3256	244
	3257	248
	3264	230
Day2	2839	481
	3002	534
	2992	508
	2985	498
	3144	521
Day3	2972	528
	3086	551
	2981	490
	2974	529
	2870	520

Table S6. Key experimental details for image analysis

Section	Details
**Cell Size Calculation (in Pixels)**	
Jurkat Cells (Diameter)	- 10–15 μm
Conversion Formula (for 10 μm)	- Pixels for 10 μm = 10 × Pixels per micrometer
Conversion Formula (for 15 μm)	- Pixels for 15 μm = 15 × Pixels per micrometer
A549 Cells (Diameter)	- 14.93 μm and 10.59 μm
**Image Processing Guidelines**	
1. Background Subtraction	- Use "Process > Subtract Background" in ImageJ to correct uneven illumination before thresholding.
2. Noise Reduction	- Apply "Process > Filters > Median" to reduce noise before analysis.
**Experimental Details**	
1. Number of Replicates	- 3 different concentrations (50 ng, 100 ng, 200 ng) with 5 microfluidic chips per concentration.<br>- Each chip has duplicate concentrations, resulting in 10 replicates per concentration.
2. Image Capturing	- Imaged at 10x magnification with 3 captures per filter.<br>- Resolution adjusted for 3D cultures.
3. Number of Images Processed	- Cell counts conducted on 5 images per chip, with manual counting for high-concentration scenarios.
4. Number of Areas Analyzed	- Each ROI counted ~5 times, results calculated separately for time points (0-48 hours).
5. Image Processing Steps	- Each ROI counted ~5 times, results calculated separately for time points (0-48 hours).

Movies

Movie S1.
Biochip gradient stimulator structure.

Movie S2.
Jurkat cells were cultured after 16 hours in RPMI+10%FBS+IL-6. CellTracker™ Red CMTPX Dye was used.
